# Case Report: Genomic profiling in an invasive solid papillary carcinoma patient with liver metastasis and a history of invasive lobular carcinoma

**DOI:** 10.3389/pore.2025.1612129

**Published:** 2025-09-09

**Authors:** Xuan Wang, Feng Zhu, Hui Wang, Shaojie Sheng, Tongbing Chen

**Affiliations:** Department of Pathology, The Third Affiliated Hospital of Soochow University, Changzhou, Jiangsu, China

**Keywords:** breast cancer, solid papillary carcinoma, metastasis, next-generation sequencing, case report

## Abstract

**Introduction:**

Solid papillary carcinoma (SPC) is a rare type of breast cancer that accounts for approximately 1% of all breast cancers. Although SPC is considered an indolent tumor, metastasis occurs in a few cases. The biological behavior and genomic characteristics of invasive SPC (ISPC) need to be further explored.

**Case presentation:**

A 44-year-old woman presented with a mass in her right breast in 2016 and ultrasound-guided mammotome (MMT) vacuum-assisted biopsy (VAB) pathology indicated an invasive lobular carcinoma (ILC). The patient subsequently underwent right partial mastectomy and axillary lymph node dissection, followed by radiotherapy and hormonal therapy. Eight years later, in 2024, ultrasonography revealed a 1.3 cm*1.0 cm mixed echogenic mass in her right breast, and biopsy pathology showed solid tumor nests with mucus secretion and thin fibrovascular cores. The pathological diagnosis was SPC with positive expression of the neuroendocrine marker synaptophysin (syn). The patient underwent right subcutaneous mastectomy with prosthesis implantation, followed by hormonal therapy. Four months later, multiple masses were found in her liver by ultrasonography and contrast-enhanced magnetic resonance imaging (MRI), which were eventually confirmed as metastatic SPC by pathology. A comprehensive next-generation sequencing (NGS) panel test was performed, and more genetic changes were identified including CCND1, FGF19, GATA3, KMT2C, MEN1, TP53, BRCA2, PI3KC3, and ERCC2::KLC3 fusion. The patient was treated with hormonal therapy combined with CDK4/6 inhibitors and so far no new lesions have appeared.

**Conclusion:**

We report a case of ISPC with liver metastasis in a patient with a history of ILC. Some meaningful genetic variations were identified by NGS. Further studies are needed to elucidate the molecular characteristics of SPC and explore the best therapeutic strategies.

## Introduction

Solid papillary carcinoma (SPC) of the breast was first described in 1995 [1]. It is a rare type of breast cancer with unique clinicopathological features and is mainly seen in postmenopausal women [2]. SPC usually shows low-grade malignancy features and often exhibits neuroendocrine and mucinous differentiation [3]. SPC *in situ* is characterized by expansive solid nests with thin fibrous cores and smooth contours, whereas invasive SPC (ISPC) has ragged contours and irregular small nests on the periphery [4]. Although SPC has an indolent biological behavior, metastasis still occurs in a small number of cases [5–8]. At present, no studies have divulged the genomic characteristics of ISPC.

Thus, here, we report a case of ISPC with liver metastasis and a history of invasive lobular carcinoma to explore its clinicopathological features. A comprehensive next-generation sequencing (NGS) panel test was also conducted to investigate the genomic characteristics of ISPC.

## Case presentation

**FIGURE 1:**
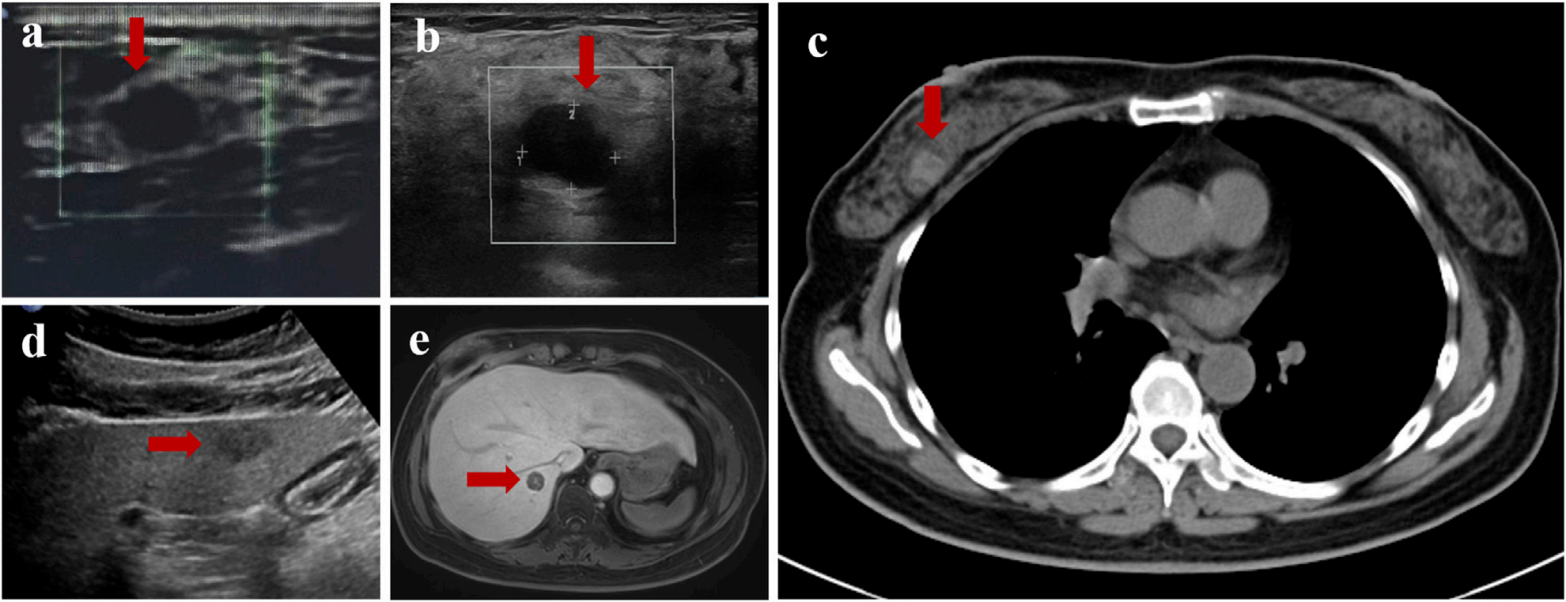
Imaging findings of this case. **(a)** Ultrasonography revealed a low-density mass measuring 1.1 cm* 0.8 cm in the right breast in 2016. **(b)** Ultrasonography revealed a 1.3 cm*1.0 cm mixed echogenic mass with clear boundaries in the right breast in 2024. **(c)** Computed tomography (CT) showed a high-density mass measuring 1.4 cm in the right breast in 2024. **(d)** The largest hypoechoic mass the abdominal ultrasound showed in the liver in 2024, measuring 1.6*1.4 cm in size. **(e)** The largest round-shaped abnormal signals displayed by magnetic resonance imaging (MRI), with a diameter of 1.8 cm.

**FIGURE 2:**
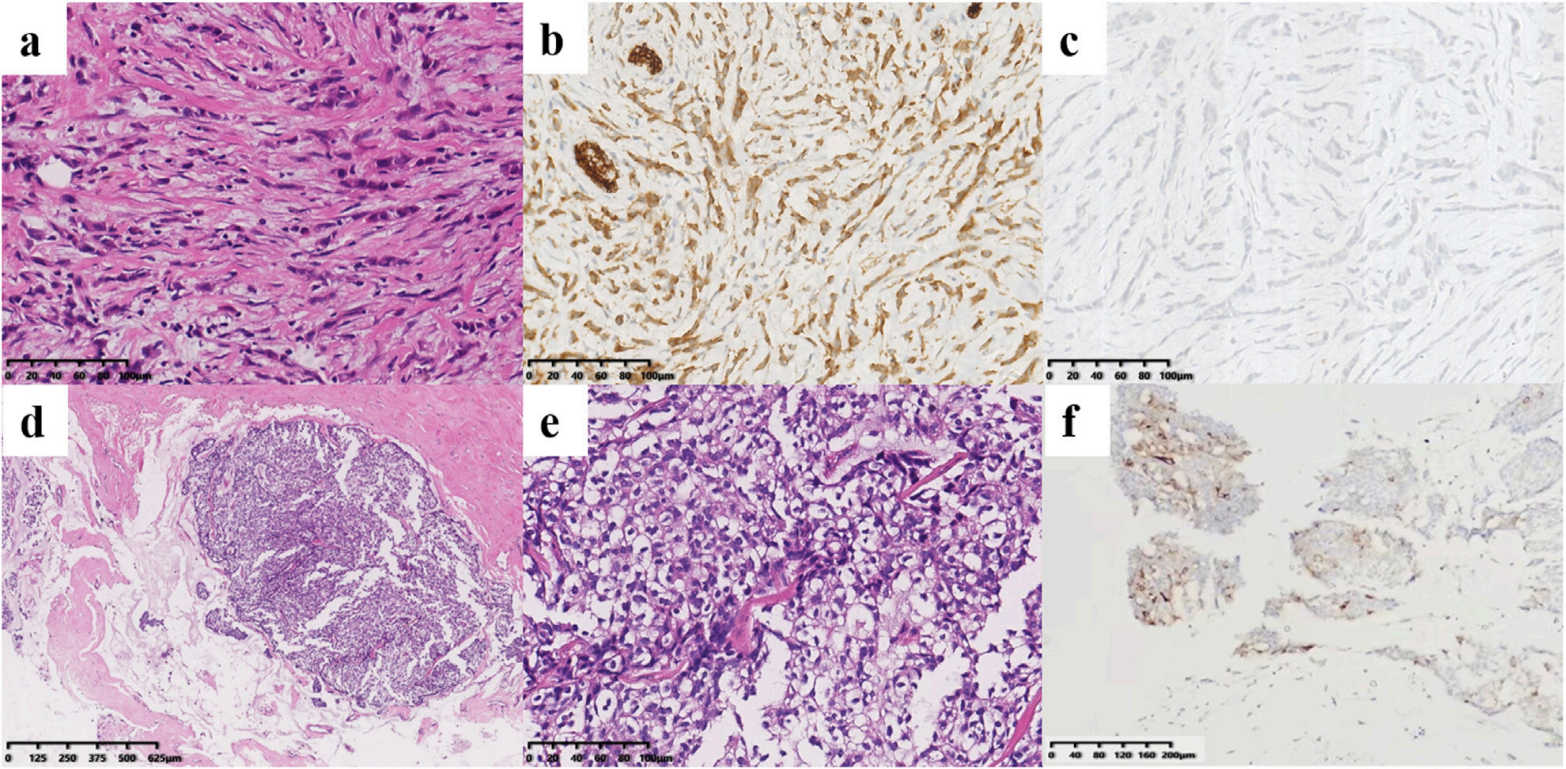
Histological findings of the right breast masses. **(a–c)** Pathology of the ILC. **(a)** Tumor cells of ILC infiltrated in cords in the connective tissue matrix (×200). They were cytoplasmic positive for P120 [**(b)**, ×200] but negative for E-cadherin [**(c)**, ×200]. **(d–f)** Pathology of the SPC. The tumor cells were oval, mildly atypical and arranged in solid nests with thin fibrovascular cores, and some irregular tumor cell nests can be seen in the mucus [**(d)**, ×40; **(e)**, ×200]. **(f)** The SPC tumor cells were positive for the neuroendocrine marker Syn (×100).

**FIGURE 3:**
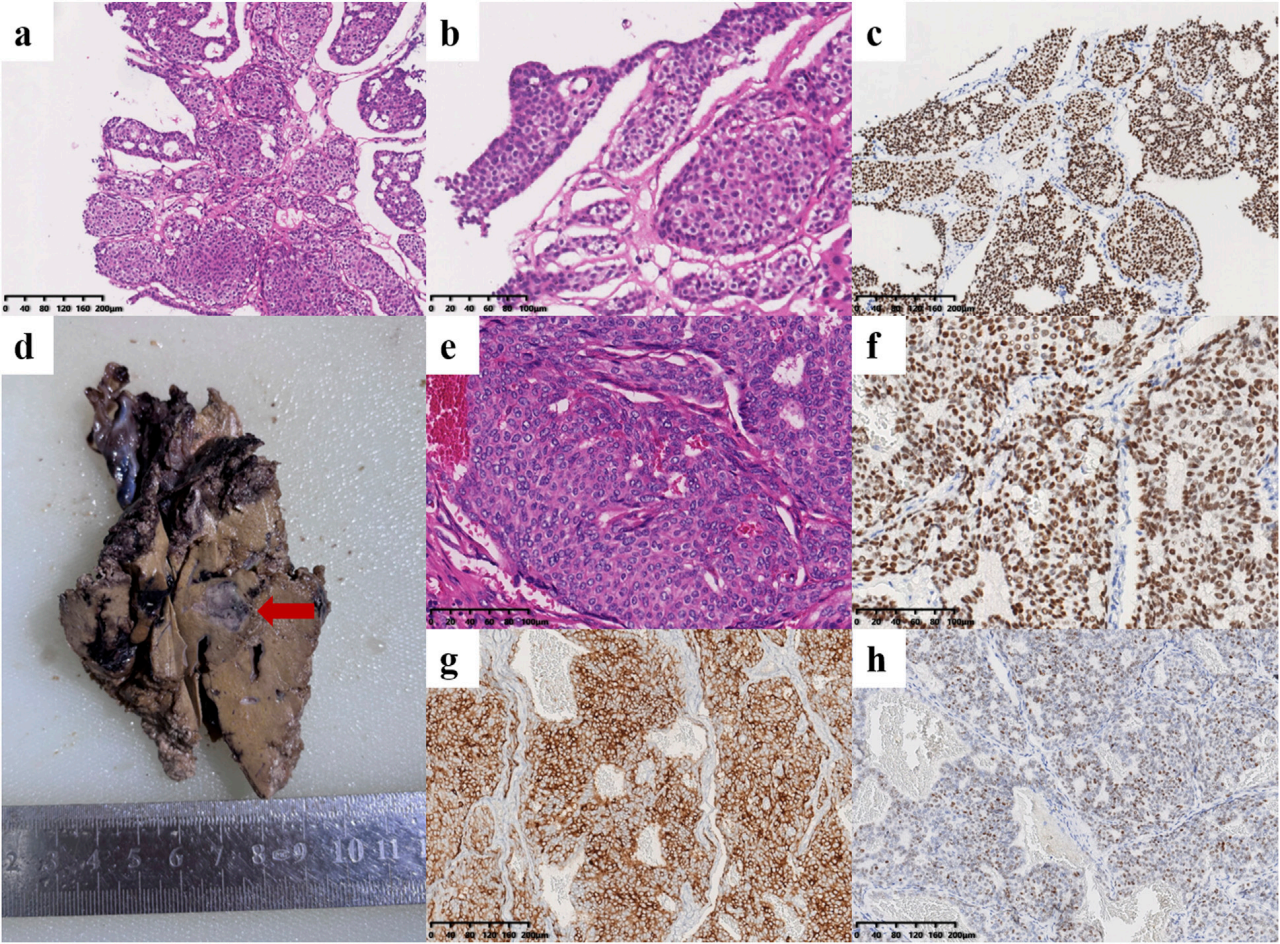
Histological findings of the liver metastases. **(a–c)** Pathology of core needle biopsy. The tumor cells were arranged in a solid nest or cribriform pattern with low atypia [**(a)**, ×100; **(b)**, ×200]. **(c)** Immunohistochemical staining revealed that the tumor cells were positive for TRPS1 (×100). **(d–h)** Pathology after partial hepatectomy. **(d)** One of the tumors in the postoperative surgical specimen of the liver (red arrow). **(e)** Histopathological examination showed solid tumor nodules with oval nuclei of uniform size (×200). **(f)** The tumor cells were positive for TRPS1 (×200). The neuroendocrine markers Syn [**(g)**, ×100] and INSM1 [**(h)**, ×100] were positive.

A 44-year-old female patient underwent breast ultrasonography, and a low-density mass (BI-RADS 3) measuring 1.1 cm*0.8 cm was found in her right breast (Figure 1a). She was admitted to our hospital in July 2016 and underwent ultrasound-guided Mammotome (MMT) vacuum-assisted biopsy (VAB). Biopsy pathology revealed invasive lobular carcinoma (ILC) with tumor cells arranged in a single-file pattern of cords (Figure 2a). The clinical stage was stage IA (cT1N0M0). Immunohistochemical staining results (Table 1) indicated that the tumor cells were positive for estrogen receptor (ER) (80%, 2+) and progesterone receptor (PR) (60%, 2+) (Supplementary Material 1). Human epidermal growth factor receptor-2 (HER2) was negative. P120 (Figure 2b) was cytoplasmic positive while E-cadherin (Figure 2c) was negative. CK34βE12 was positive while CK5/6 was negative. The Ki-67 index was 10%. The patient subsequently underwent right partial mastectomy and axillary sentinel lymph node dissection, and no lymph node metastasis was found. After the operation, the patient received radiotherapy and hormonal therapy and underwent regular breast ultrasound examination. No recurrence of the disease was reported until May 2024, when ultrasonography revealed a 1.3 cm*1.0 cm mixed echogenic mass with clear boundaries in the right breast without enlarged axillary lymph nodes (Figure 1b). She subsequently underwent a chest computed tomography (CT) scan, which showed a high-density mass measuring 1.4 cm in the right breast (Figure 1c). An ultrasound-guided MMT VAB revealed solid tumor nests with thin fibrovascular cores and some irregular cell nests floating in the mucus (Figure 2d). The tumor cells were mildly atypical with oval nuclei and ample cytoplasm (Figure 2e). The pathological diagnosis was ISPC rather than ILC, and immunohistochemistry was performed to confirm it (Supplementary Material 2). The tumor cells were positive for ER (80% 2+), PR (50% 1+), synaptophysin (syn) (Figure 2f) and E-cadherin but negative for chromogranin A (CgA), the myoepithelial markers p63 and calponin. The expression of HER2 protein was low (1+). According to the above examination results, the clinical stage of the patient was stage IA (cT1N0M0). The patient subsequently underwent right subcutaneous mastectomy with prosthesis implantation. Hormonal therapy (Arimidex and a gonadotropin-releasing hormone (GnRH) agonist) was applied as adjuvant therapy on the basis of the pathological diagnosis. Four months later, the patient’s abdominal ultrasound showed multiple hypoechoic masses in the liver, the largest of which was 1.6*1.4 cm in size (Figure 1d). Unfortunately, these masses were not present on the CT scan 4 months ago. Magnetic resonance imaging (MRI) demonstrated that there were multiple round-shaped abnormal signals in the liver, the largest of which was 1.8 cm in diameter (Figure 1e). Core needle biopsy (CNB) pathology showed that the tumor cells were arranged in a solid nest or cribriform pattern with pale eosinophilic cytoplasm and oval nuclei of uniform size (Figures 3a, b). In the immunohistochemical analysis (Supplementary Material 3), the tumor cells were positive for GATA-binding protein 3 (GATA3) and trichorhinophalangeal syndrome type 1 (TRPS1) (Figure 3c) but negative for HepPar1 and Glypican-3, which indicated that the tumor originated in the breast rather than the liver. ER was strongly positive (90% 2–3+) while PR was negative. The score of HER2 was 1+, and the Ki-67 index was 2%. The neuroendocrine marker Syn was positive while CgA was negative, further confirming that breast SPC had metastasized to the liver. Subsequently, the patient underwent partial hepatectomy, and visible tumors were found in the surgical specimen (Figure 3d). Postoperative pathology revealed a solid papillary growth pattern (Figure 3e), and the pathological diagnosis was also metastatic breast SPC (Supplementary Material 4). The tumor cells were positive for GATA3, TRPS1 (Figure 3f), E-cadherin and the neuroendocrine markers Syn (Figure 3g) and insulinoma associated protein 1 (INSM1) (Figure 3h). ER was positive (80% 2+) but PR was negative. The score of HER2 was 1+, and the Ki-67 index was approximately 3%. Considering that distant metastasis of SPC is relatively rare, a comprehensive next-generation sequencing (NGS) panel test (provided by Nanjing Shihe Medical Laboratory Co., Ltd.) involving 481 genes was conducted on the liver metastases (Supplementary Material 5). After observing the HE-stained sections of the specimens under the microscope, we select paraffin-embedded tissue sections for NGS testing that must contain a sufficient proportion of tumor cells (in this case, the sample is 70%) and a sufficient number of tumor cells, while trying to eliminate non-tumor cells, necrosis, and mucus on the corresponding white sections, and avoiding cross-contamination between different case tissues. For the formalin-fixed paraffin-embedded samples, ten 5 μm tumor slices were used for DNA extraction using the QIAamp DNA FFPE Kit (QIAGEN, Valencia, CA, USA) following the manufacturer’s instructions. DNA quality was assessed by spectrophotometry with absorbance at 230,260, and 280 nm, and quantified by Qubit 2.0. Sequencing data were mapped to the reference hg19 genome (Human Genome version 19) using the Burrows-Wheeler Aligner [9]. The Genome Analysis Toolkit (GATK) [10] was used to perform local realignments around indels and base quality reassurance. Hotspot SNPs and indels were called by VarScan2 [11] and HaplotypeCaller/UnifiedGenotyper in GATK, with the mutant allele frequency (MAF) cutoff as 0.4% (1% for non-hotspot) and a minimum of ten (six for non-hotspot) unique mutant reads for tissue samples. The resulting somatic variants were further filtered through an in-house list of recurrent sequencing errors that was generated from over 10,000 normal control samples on the same sequencing platform. Gene fusions were identified by FACTERA [12] and copy number variations (CNVs) were analyzed with ADTEx [13, 14]. This analysis identified several genetic alterations including copy number gain of CCND1 and FGF19, copy number loss of TP53, a fusion between ERCC2 and KLC3 (Supplementary Material 6), and mutations of GATA3, KMT2C, MEN1, BRCA2, and PIK3C3, which are displayed in Table 2. After partial hepatectomy in September 2024, the patient was treated with hormonal therapy combined with CDK4/6 inhibitors, and no new lesions have been found thus far.

**TABLE 1:** Immunohistochemical staining results of ILC, invasive SPC and metastatic SPC in the patient.

Tumor types Markers	ILC	Invasive SPC	Metastatic SPC in the liver	
			Biopsy specimen	Postoperative surgical specimen
ER	80% 2+	80% 2+	90% 2–3+	80% 2–3+
PR	60% 2+	50% 1+	-	-
HER2	0	1+	1+	1+
E-cadherin	-	+	+	+
P120	cytoplasmic +	—	—	—
CK5/6	-	-	—	—
CK34βE12	+	—	—	—
Calponin	—	-	—	—
P63	—	-	—	—
CgA	—	-	-	-
Syn	—	+	+	+
INSM1	—	—	—	+
GATA3	—	—	+	+
TRPS1	—	—	+	+
HepPar1	—	—	-	-
Glypican-3	—	—	-	-
Ki-67	10% +	10% +	2% +	3% +

**TABLE 2:** Somatic gene alterations by next-generation sequencing (NGS).

Gene	Variation type	Classification^a^	Frequency (%)
CCND1	Copy number gain	VUS: uncertain significance	CN: 7.38
FGF19	Copy number gain	no record	CN: 5.36
GATA3	c.1321dup (p.T441Nfs^*^67)	no record	32.41%
KMT2C	c.910C>T (p.Q304^*^)	no record	31.88%
MEN1	c.1174del (p.E392Sfs^*^53)	pathogenic/likely pathogenic	54.06%
TP53	Copy number loss	no record	CN: 1.11
BRCA2	c.4307T>C (p.I1436T)	conflicting classification of pathogenicity: uncertain significance/likely benign	18.01%
ERCC2	KLC3: exon8∼ERCC2: exon23	no record	65.11%
PI3KC3	c.368G>A (p.G123E)	no record	37.42%

Abbreviation: VUS, variants of uncertain significance.

^a^ The classifications of these gene alterations were searched in ClinVar database (https://www.ncbi.nlm.nih.gov/clinvar/). And “no record” means that we did not find this record in the database.

The patient is the sole individual in her family diagnosed with cancer, with no family history among her relatives.

## Discussion

SPC is considered an indolent tumor that rarely metastasizes or causes death [15]. In the present case, the patient was first diagnosed with invasive lobular carcinoma of her right breast in 2016. Unfortunately, she found a mass in her right breast again in 2024 and was diagnosed with SPC. The clinical stage was stage IA (cT1N0M0) until liver metastasis was found 4 months later, and the clinical stage became stage IV. The timeline is presented in Table 3. Some genetic changes including some tumor susceptibility genes or driver genes such as CCND1, FGF19, GATA3, KMT2C, MEN1, TP53, BRCA2, PI3KC3 and a fusion between ERCC2 and KLC3, were identified via NGS test of the liver metastasis tissue.

**TABLE 3:** Timeline.

Time	Imaging finging	Pathological diagnosis
July 2016	Ultrasonography showed a mass in the right breast	ILC
May 2024	Ultrasonography and CT scan revealed a new mass in the right breast	Invasive SPC
September 2024	Ultrasonography and MRI revealed multiple masses in the liver	Metastatic SPC

Most of the genetic alterations identified in this study are common in breast cancer, such as TP53 (44%–48.6%) [16, 17], CCND1 (11%–12.9%) [16, 18], FGF19 (8%–30%) [16, 17, 19], GATA3 (8%–11%) [16, 17], KMT2C (8.8%) [20] and BRCA2 (5%–14%) [16, 19]. Papillary carcinomas of the breast display relatively simple genomic profiles, and exhibit the genomic features similar to low-grade ER-positive breast cancers, including 16q losses, 16p gains and 1q gains [21, 22]. 11q13.3 gains were also found in 12% of breast papillary carcinomas, encompassing CCND1 [23], and copy number gain of CCND1was also identified in our study.

The CCND1 gene encodes the cyclinD1 protein, which plays a crucial role in regulating cell cycle. The rate of CCND1 amplification is 12.9% in breast cancer, most which are luminal B (51.5%) or luminal A subtype (25.8%) [18]. In addition, CCND1 amplification predicted shorter RFS (recurrence-free survival) and OS (overall survival) in patients treated with endocrine therapy [24].

Somatic variation in BRCA 2 (p.I1436T) was detected in this patient’s tumor. BRCA1/2 are the most common genes related to breast cancer susceptibility [25], and germline BRCA mutations (gBRCAm) lead to hereditary breast cancer and/or ovarian cancer (HBOC) syndrome [26]. Triple-negative breast cancer patients are more likely to carry BRCA1/2 mutations than hormone receptor-positive patients are, as evidenced by the proportions of 16% and 4%, respectively [27]. However, the probabilities of BRCA1 and BRCA2 mutations in breast SPC has never been reported. The proteins encoded by BRCA1 and BRCA2 play critical roles in DNA damage repair by mediating homologous recombination (HR) [28]. BRCA1/2 mutant breast cancer cells are more sensitive to poly (ADP-ribose) polymerase (PARP) inhibitors because of the synthetic lethal mechanism [29], and olaparib has been approved for the adjuvant treatment of gBRCAm, HER2-negative high-risk early breast cancer and gBRCAm, HER2-negative locally advanced or metastatic breast cancer [30]. Our study identified somatic mutations of BRCA2 in an ISPC, hoping to provide useful information for the adjuvant treatment of ISPC in the future.

The MEN1 gene that encodes menin protein is located on 11q13.1, and usually undergoes germline inactivating mutations result in multiple endocrine neoplasia type 1 [31]. Patients with multiple endocrine neoplasia type 1 are reported to have a significantly elevated breast cancer risk [32]. *In vitro* experiments indicated that menin protein directly interacted with the AF-2 domain of ER-alpha and enhances ER activity in breast cancer progression [33]. However, Tamoxifen inhibited the interaction between menin and ER-alpha, and over-expression of menin caused tamoxifen resistance in a clinical study with 65 tamoxifen-treated ER-positive breast cancer samples [33]. Frameshift mutation of MEN1 gene was identified in our case, unfortunately we did not further examine menin protein expression of the tumor. Further researches of MEN1 gene and menin protein may contribute to the therapeutic strategies of breast cancer.

KMT2C/D plays a tumor-suppressive role in the development of breast cancer, and promotes ER-driven transcription in ER positive breast cancer by activating gene enhancer region [34]. FGFR19 binds to FGFR4 to transfer endocrine signaling, and FGF19-FGFR4 signaling is closely associated with cancer development and progression [35]. Although alterations in KMT2C and FGF19 are common in breast cancer, there is currently no clinical evidence of the correlation between them and the therapeutic effect of breast cancer.

Notably, a fusion between ERCC2 and KLC3 was also detected in this case. ERCC2 encodes xeroderma pigmentosum group D, and is involved in the nucleotide excision repair pathway [36]. A previous study showed that among women with second primary cancers, the pathogenic variants of BRCA1 (HR = 2.28, 95% CI = 1.11–4.65) and ERCC2 (HR = 3.51, 95% CI = 1.29–9.54) were significantly enriched, and pathogenic variants of ERCC2 (HR = 5.09, 95% CI = 1.58–16.4) were significantly associated with second primary breast cancers [37]. We present a case of second primary breast SPC in a patient with a history of ILC, and a fusion between KLC3 exon 8 and ERCC2 exon 23 was identified, which has not been reported in previous studies. Although no research has divulged the significance of ERCC2 in the prognosis and treatment of breast cancer, ERCC2 mutation was reported to be an independent predictor in bladder cancer patients and a potential marker for chemosensitivity in primary and secondary muscle-invasive bladder cancer patients [38, 39]. Perhaps further studies on the significance of ERCC2 variations in breast cancer patients will emerge in the future.

## Conclusion

In conclusion, we reported a case of ISPC with liver metastasis in a patient with a history of ILC. NGS was conducted, and some significant genetic variations were identified, revealing insights into the genetic characteristics of and adjuvant treatment for ISPC. Further research on the biological behavior and molecular characteristics of ISPC is needed.

## Data Availability

The original contributions presented in the study are included in the article/Supplementary Material, further inquiries can be directed to the corresponding author.

## Supplementary material

SUPPLEMENTARY DATA SHEET 1 IHC pictures of breast ILC.

SUPPLEMENTARY DATA SHEET 2 IHC pictures of breast SPC.

SUPPLEMENTARY DATA SHEET 3 IHC pictures of liver biopsy specimen.

SUPPLEMENTARY DATA SHEET 4 IHC pictures of liver postoperative surgical specimen.

SUPPLEMENTARY DATA SHEET 5 Raw data of NGS.

SUPPLEMENTARY IMAGE 1 ERCC2::KLC3 fusion.

## Abbreviations

BRCA1/2, breast cancer 1/2; CCND1, cyclin D1; CgA, chromogranin A; CNB, core needle biopsy; ER, estrogen receptor; ERCC2, excision repair cross-complementation group 2; GATA3, GATA-binding protein 3; HER2, human epidermal growth factor receptor-2; ILC, invasive lobular carcinoma; INSM1, insulinoma associated protein 1; ISPC, invasive solid papillary carcinoma; KLC3, kinesin light chain 3; MMT, mammotome; NGS, next-generation sequencing; PARP, poly (ADP-ribose) polymerase; PR, progesterone receptor; SPC, solid papillary carcinoma; Syn, synaptophysin; TRPS1, trichorhinophalangeal syndrome type 1; VAB, vacuum-assisted biopsy.
