# Supplementary figures and images for: Case Report: Genomic profiling in an invasive solid papillary carcinoma patient with liver metastasis and a history of invasive lobular carcinoma
Source: Pathol Oncol Res. 2025 Sep 9;31:1612129. doi: 10.3389/pore.2025.1612129 (PMC12454161; doi:10.3389/pore.2025.1612129)

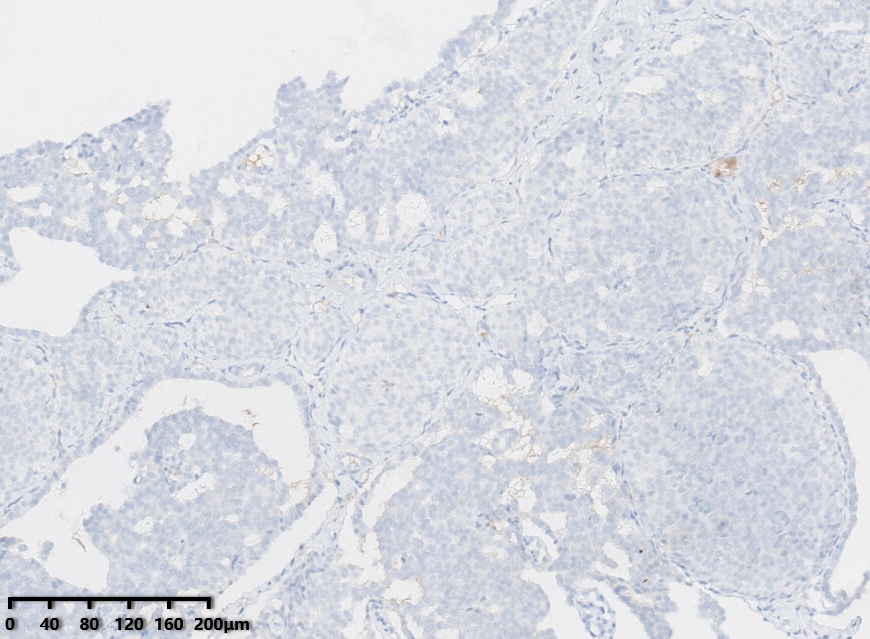

Supplement: Supplementary file 1 [file DataSheet3.zip › immunohistochemistry pictures of liver biopy specimen/CgA 100x.jpg]

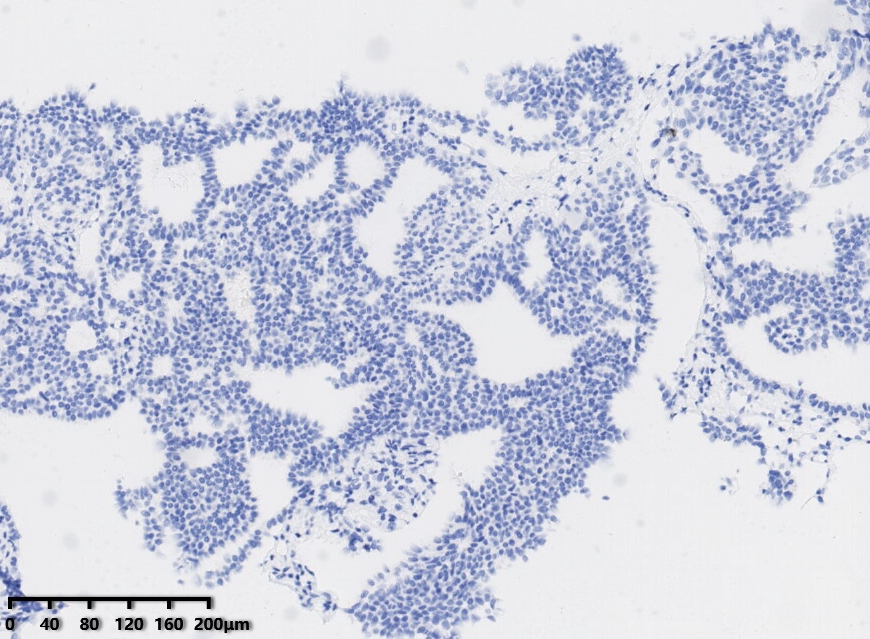

Supplement: Supplementary file 1 [file DataSheet3.zip › immunohistochemistry pictures of liver biopy specimen/CK20 100x.jpg]

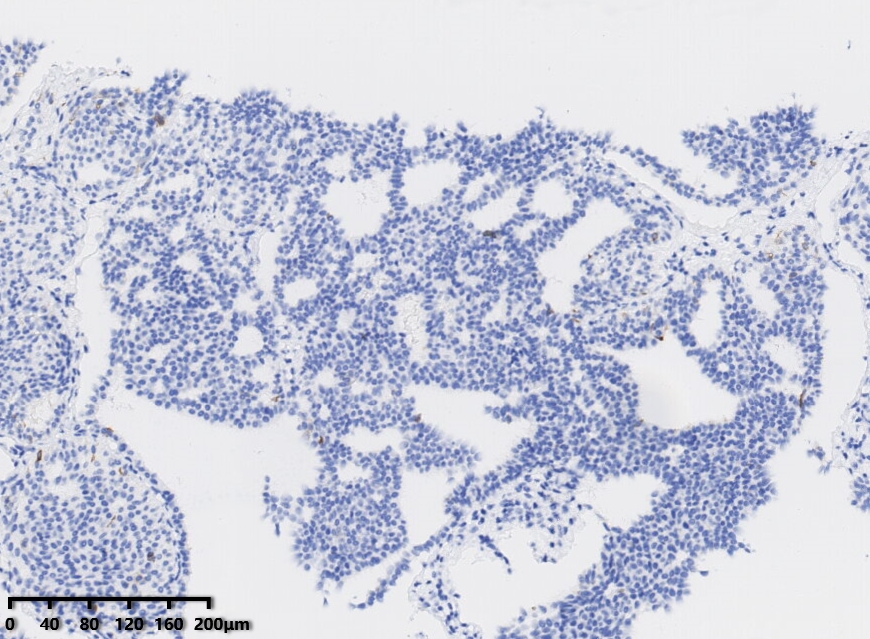

Supplement: Supplementary file 1 [file DataSheet3.zip › immunohistochemistry pictures of liver biopy specimen/CK7 100x.jpg]

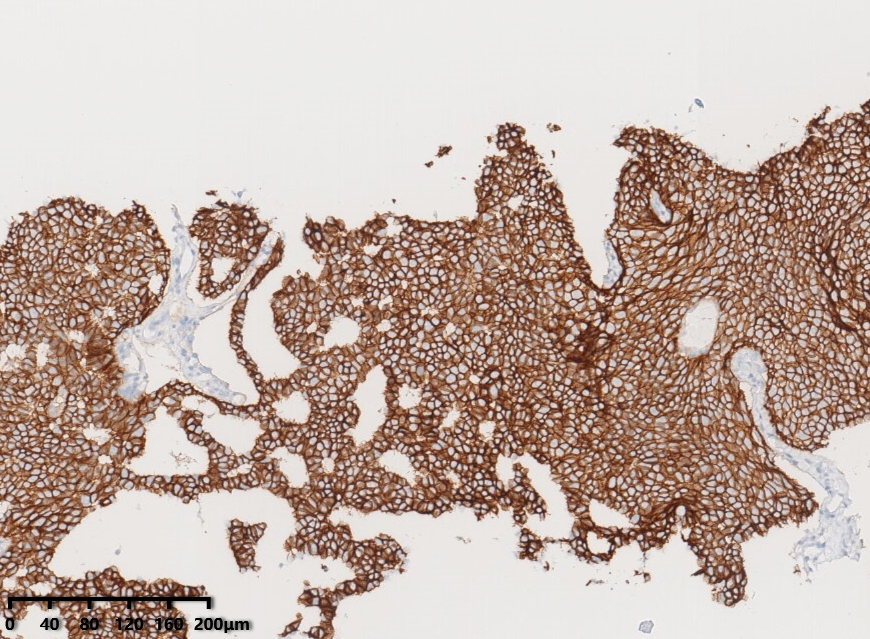

Supplement: Supplementary file 1 [file DataSheet3.zip › immunohistochemistry pictures of liver biopy specimen/E-cadherin 100x.jpg]

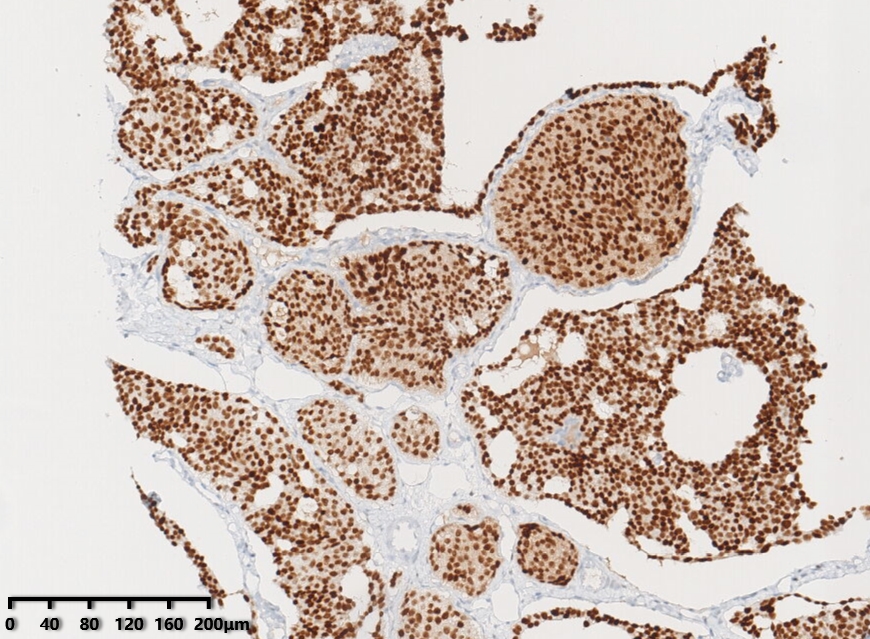

Supplement: Supplementary file 1 [file DataSheet3.zip › immunohistochemistry pictures of liver biopy specimen/ER 100x.jpg]

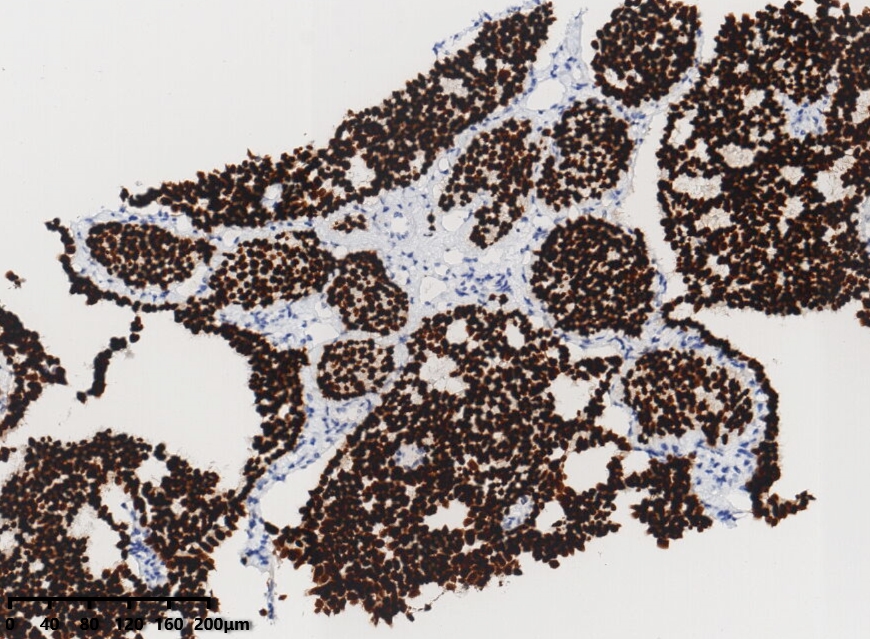

Supplement: Supplementary file 1 [file DataSheet3.zip › immunohistochemistry pictures of liver biopy specimen/GATA3 100x.jpg]

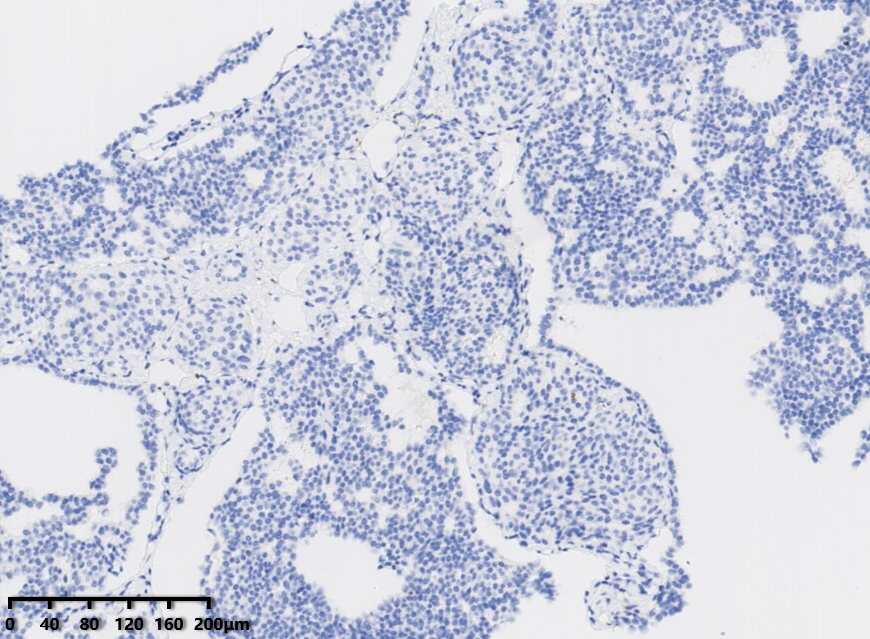

Supplement: Supplementary file 1 [file DataSheet3.zip › immunohistochemistry pictures of liver biopy specimen/GPC-3 100x.jpg]

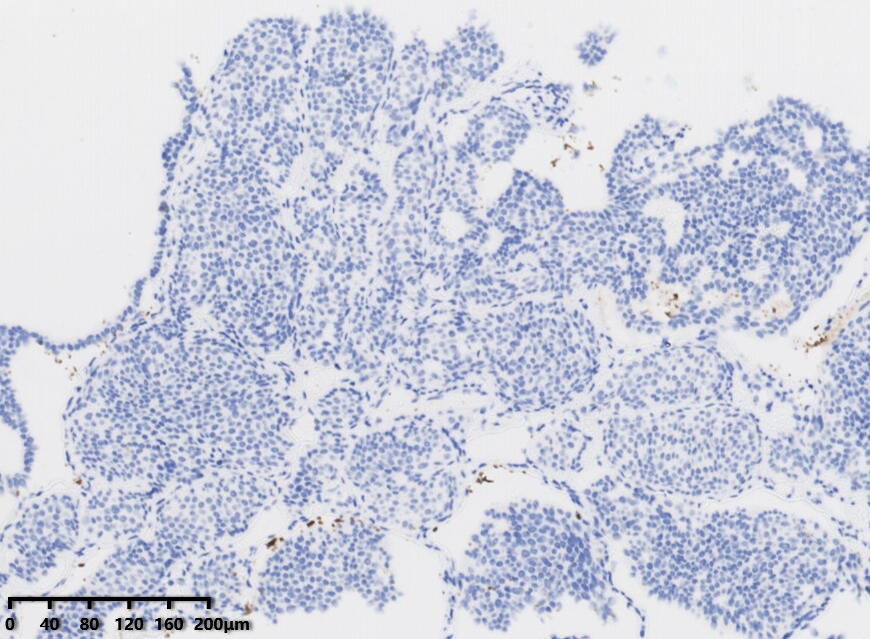

Supplement: Supplementary file 1 [file DataSheet3.zip › immunohistochemistry pictures of liver biopy specimen/hepatocyte 100x.jpg]

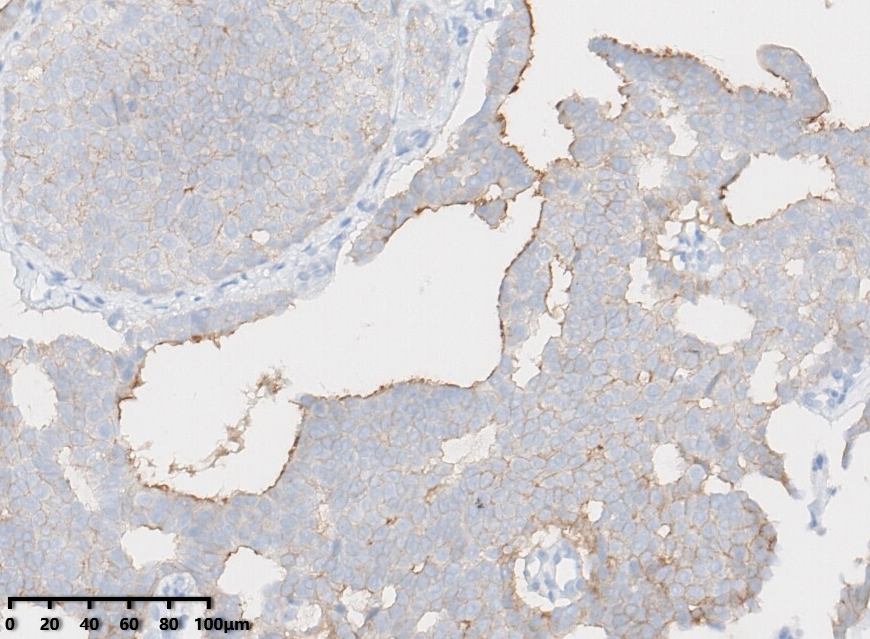

Supplement: Supplementary file 1 [file DataSheet3.zip › immunohistochemistry pictures of liver biopy specimen/HER2 200x.jpg]

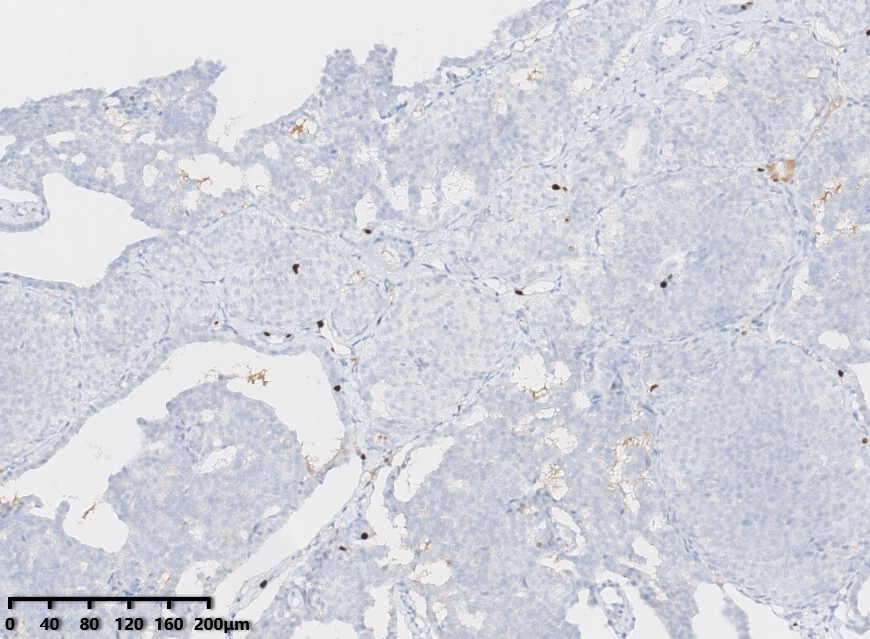

Supplement: Supplementary file 1 [file DataSheet3.zip › immunohistochemistry pictures of liver biopy specimen/Ki67 100x.jpg]

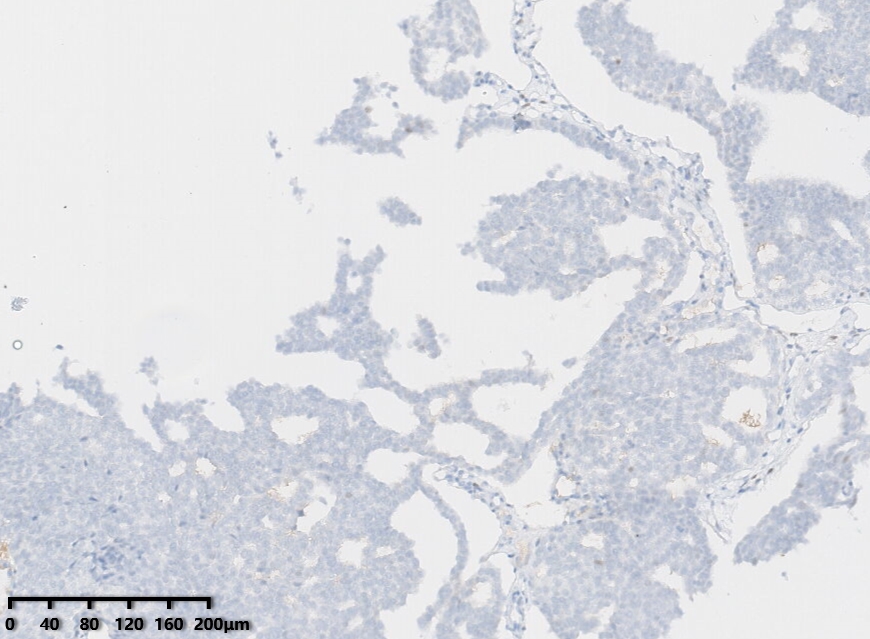

Supplement: Supplementary file 1 [file DataSheet3.zip › immunohistochemistry pictures of liver biopy specimen/PR 100x.jpg]

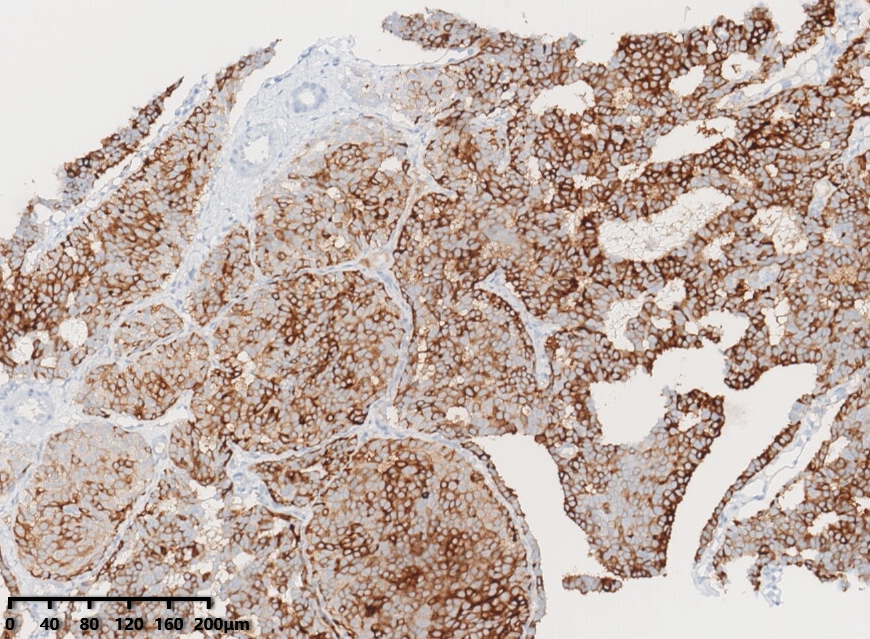

Supplement: Supplementary file 1 [file DataSheet3.zip › immunohistochemistry pictures of liver biopy specimen/Syn 100x.jpg]

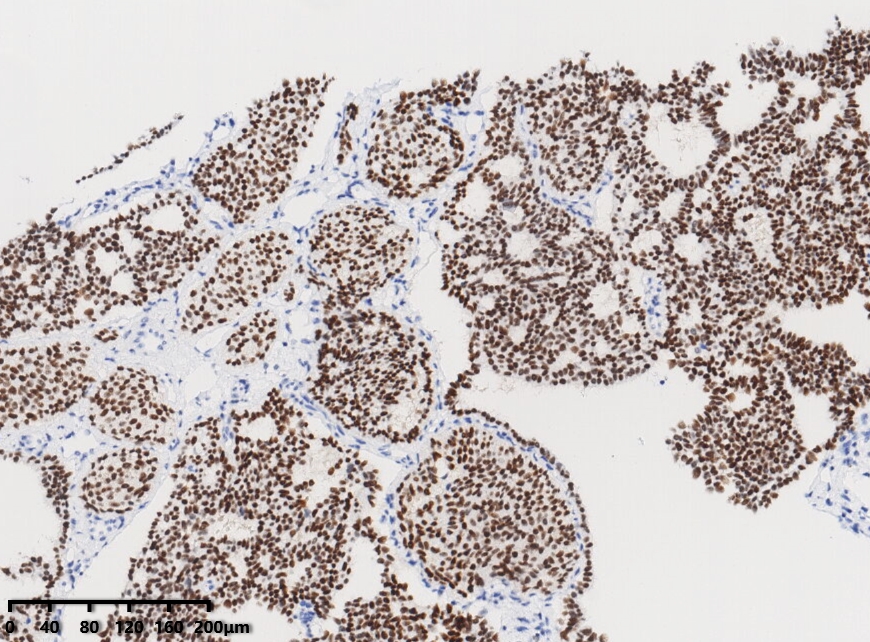

Supplement: Supplementary file 1 [file DataSheet3.zip › immunohistochemistry pictures of liver biopy specimen/TRPS1 100x.jpg]

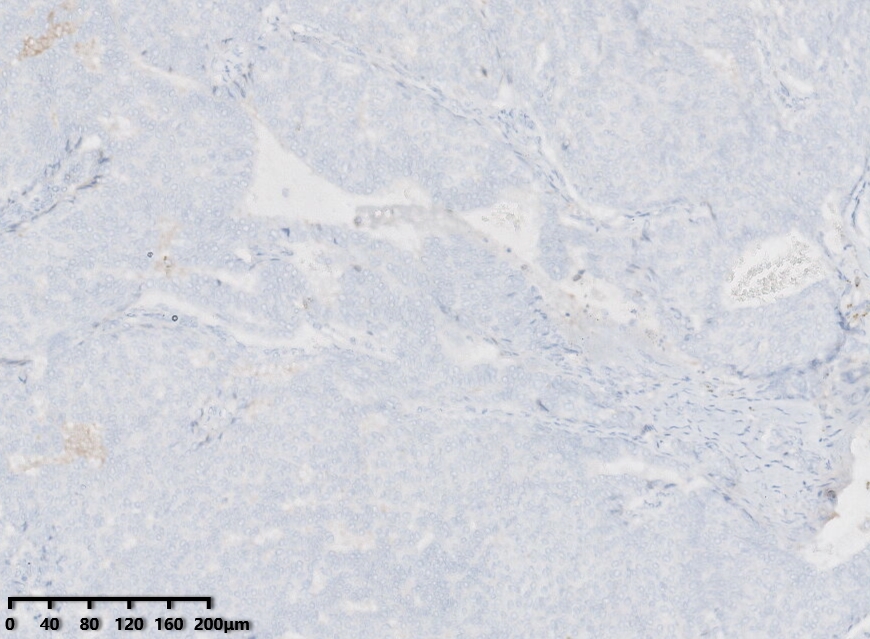

Supplement: Supplementary file 2 [file DataSheet4.zip › immunohistochemistry pictures of liver postoperative surgical specimen/CgA 100x.jpg]

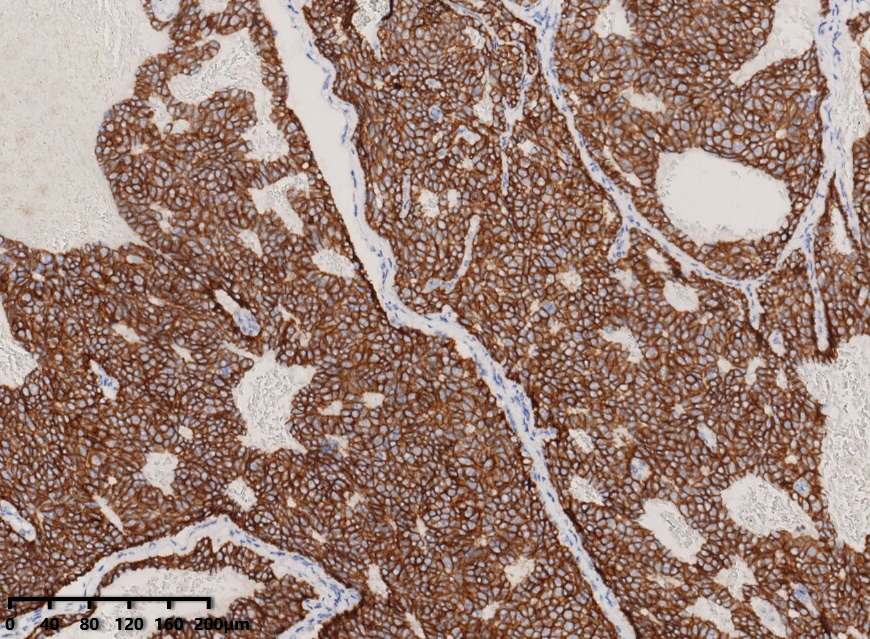

Supplement: Supplementary file 2 [file DataSheet4.zip › immunohistochemistry pictures of liver postoperative surgical specimen/E-cadherin 100x.jpg]

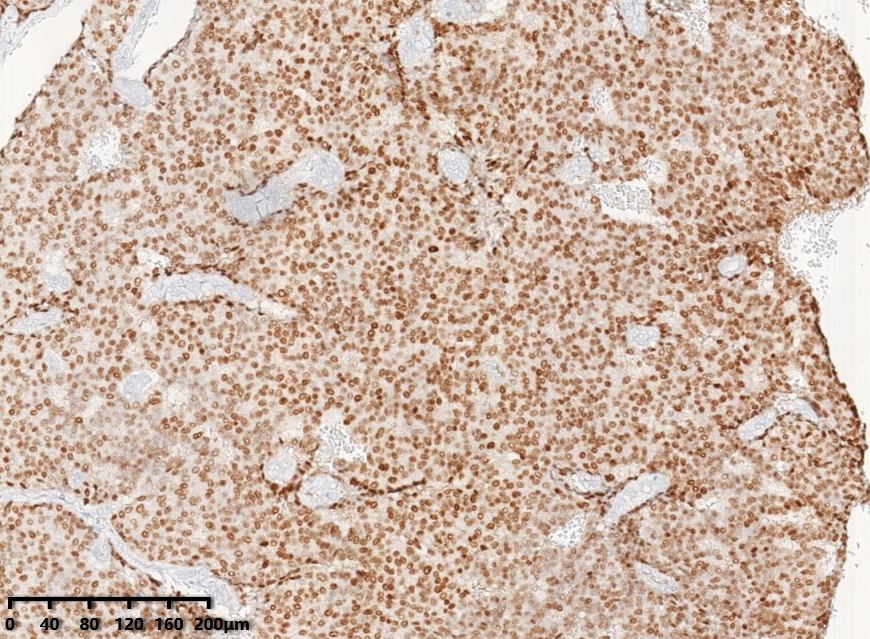

Supplement: Supplementary file 2 [file DataSheet4.zip › immunohistochemistry pictures of liver postoperative surgical specimen/ER 100x.jpg]

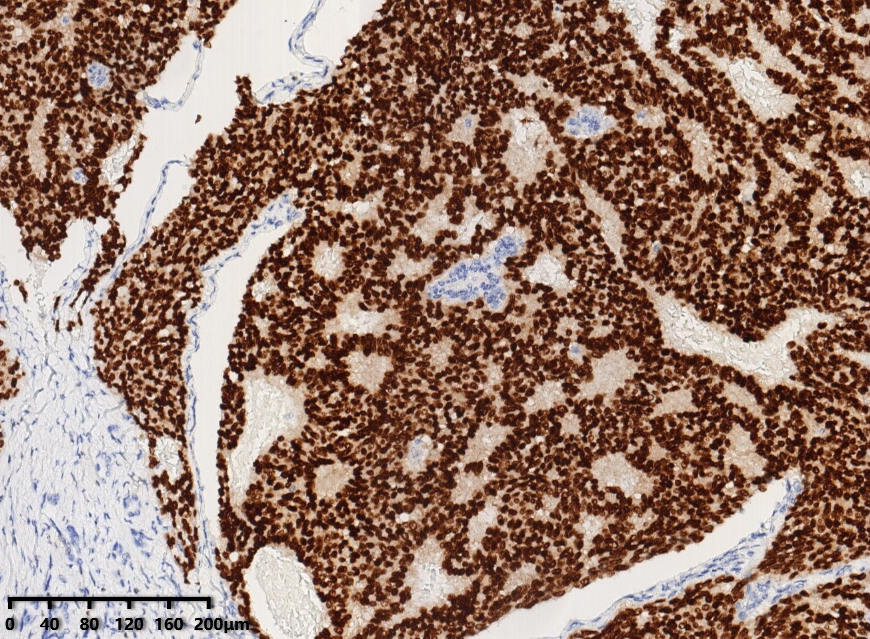

Supplement: Supplementary file 2 [file DataSheet4.zip › immunohistochemistry pictures of liver postoperative surgical specimen/GATA3 100x.jpg]

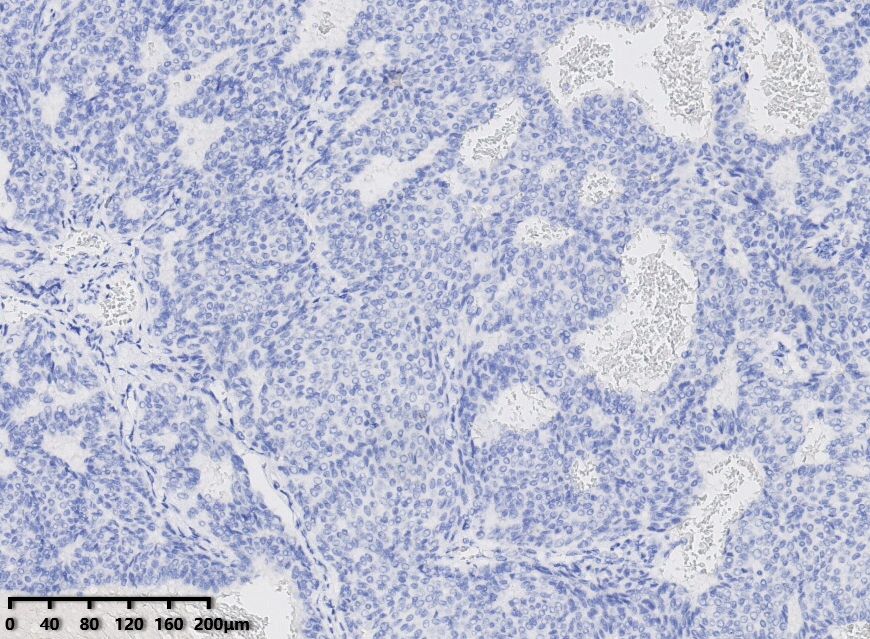

Supplement: Supplementary file 2 [file DataSheet4.zip › immunohistochemistry pictures of liver postoperative surgical specimen/Hepatocyte 100x.jpg]

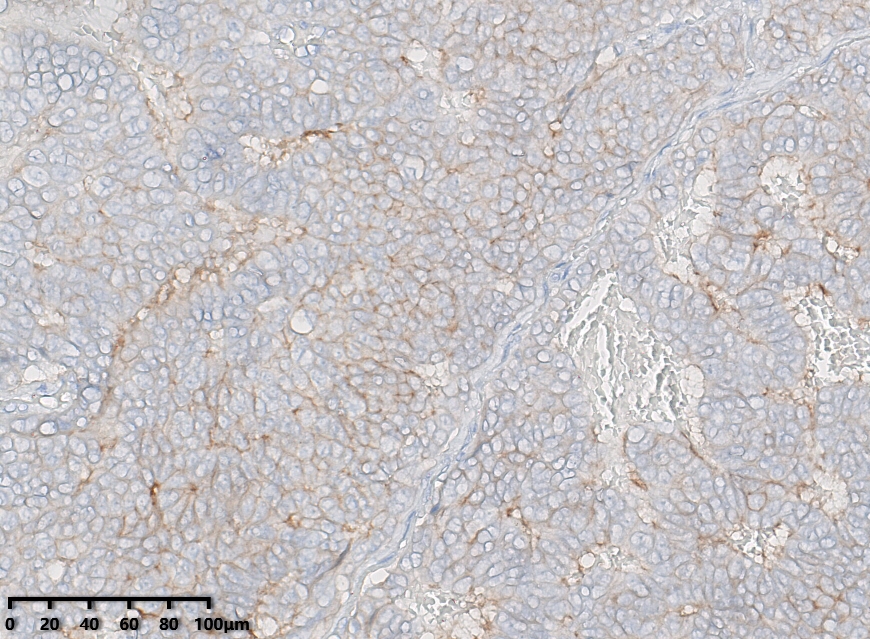

Supplement: Supplementary file 2 [file DataSheet4.zip › immunohistochemistry pictures of liver postoperative surgical specimen/HER2 200x.jpg]

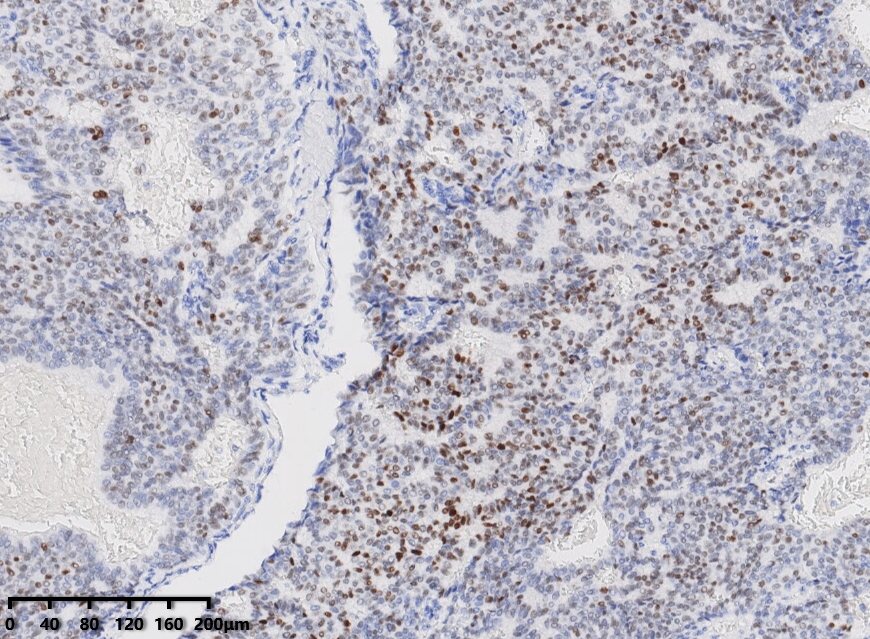

Supplement: Supplementary file 2 [file DataSheet4.zip › immunohistochemistry pictures of liver postoperative surgical specimen/INSM1 100x.jpg]

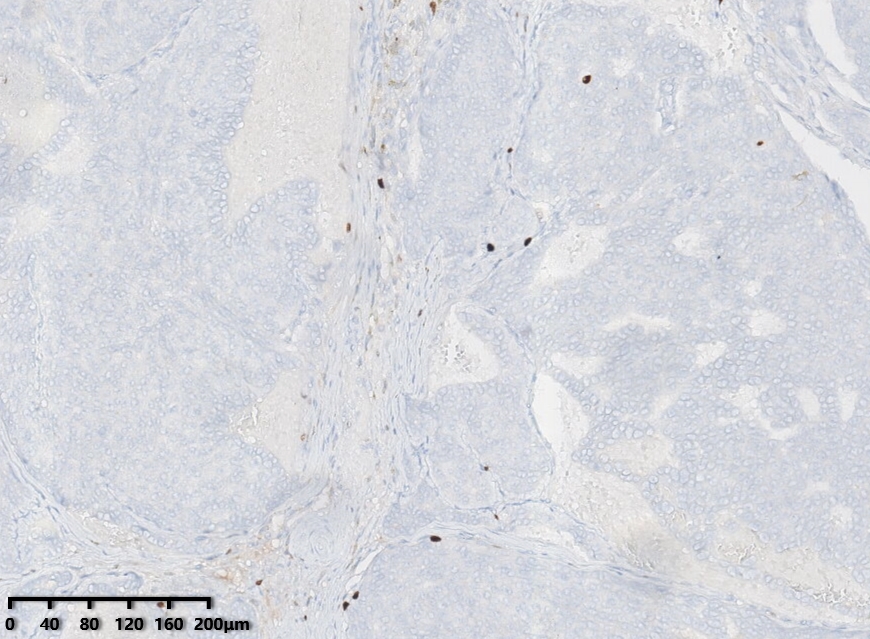

Supplement: Supplementary file 2 [file DataSheet4.zip › immunohistochemistry pictures of liver postoperative surgical specimen/Ki67 100x.jpg]

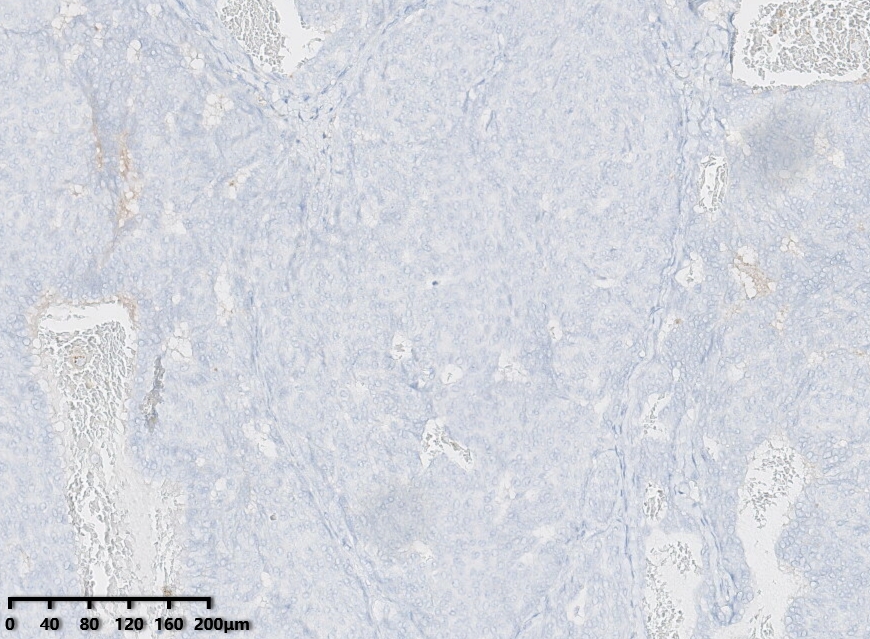

Supplement: Supplementary file 2 [file DataSheet4.zip › immunohistochemistry pictures of liver postoperative surgical specimen/PR 100x.jpg]

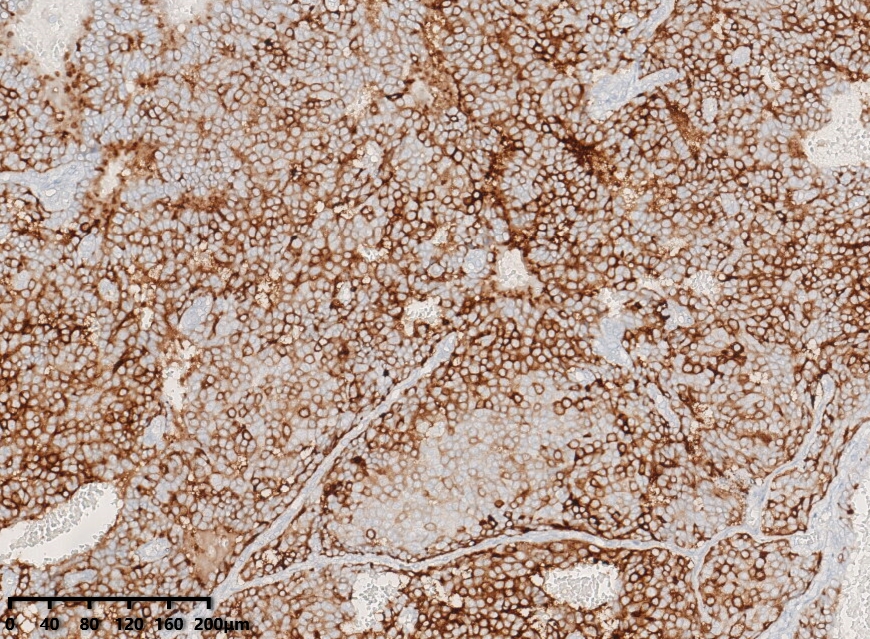

Supplement: Supplementary file 2 [file DataSheet4.zip › immunohistochemistry pictures of liver postoperative surgical specimen/Syn 100x.jpg]

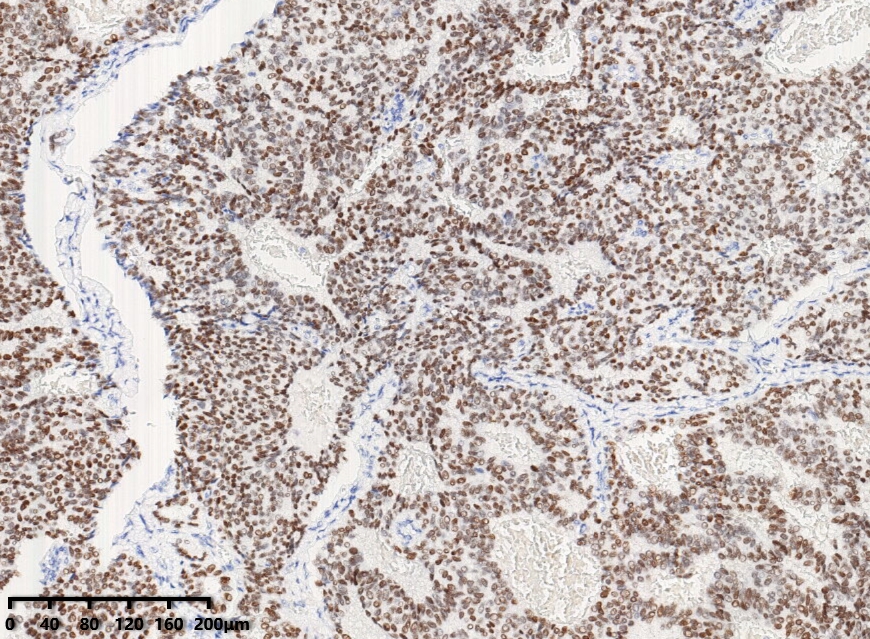

Supplement: Supplementary file 2 [file DataSheet4.zip › immunohistochemistry pictures of liver postoperative surgical specimen/TRPS1 100x.jpg]

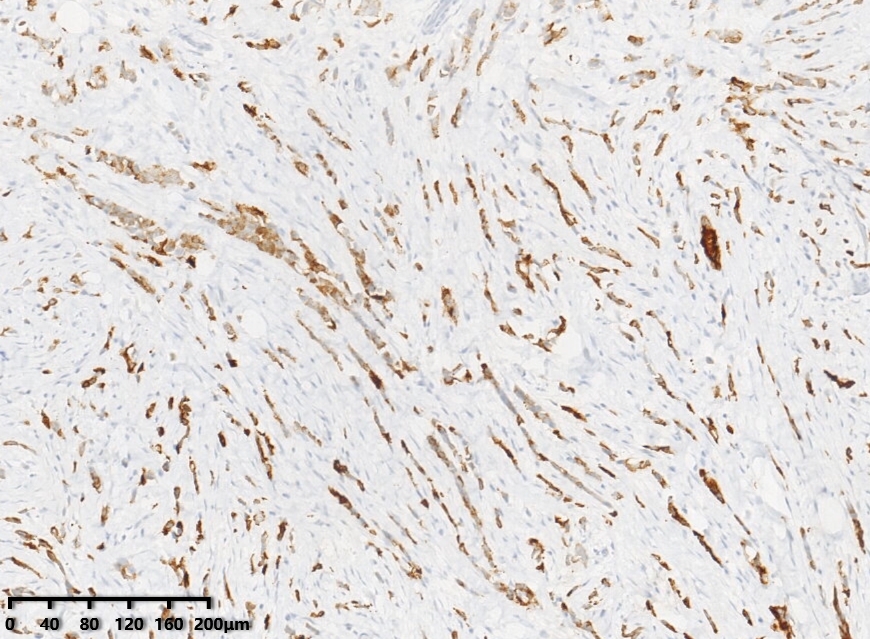

Supplement: Supplementary file 3 [file DataSheet1.zip › immunohistochemistry pictures of breast ILC/CK34βE12 100x.jpg]

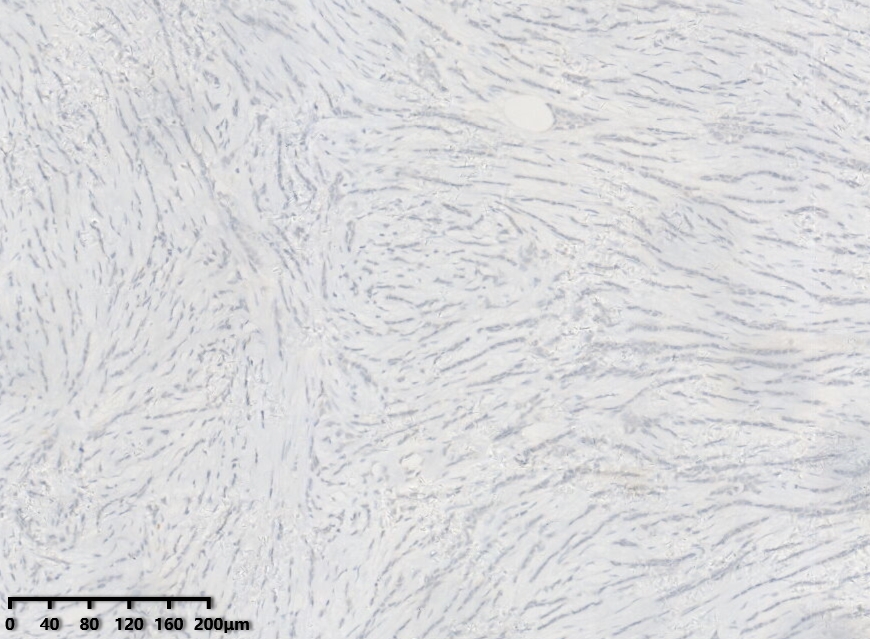

Supplement: Supplementary file 3 [file DataSheet1.zip › immunohistochemistry pictures of breast ILC/CK5&6 100x.jpg]

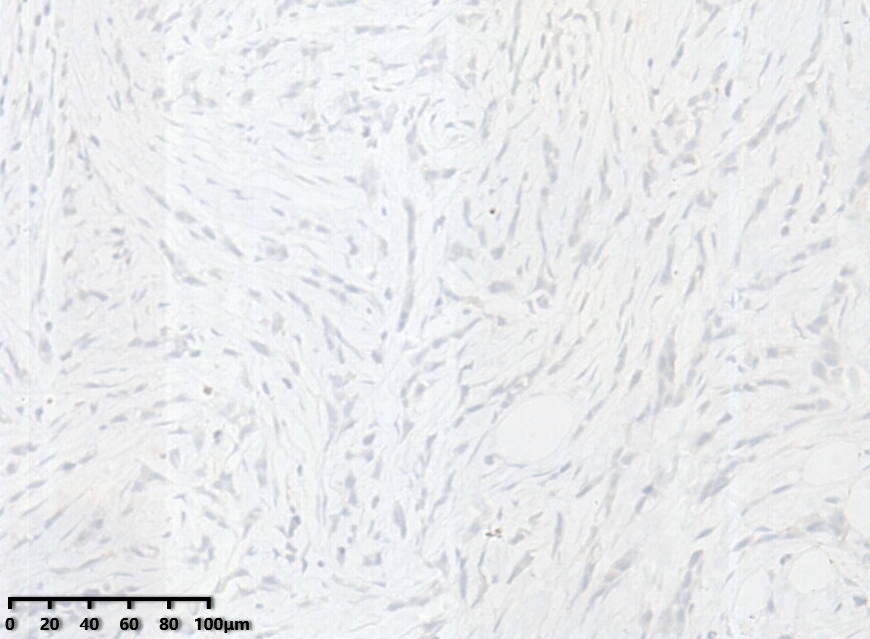

Supplement: Supplementary file 3 [file DataSheet1.zip › immunohistochemistry pictures of breast ILC/E-cadherin 200x.jpg]

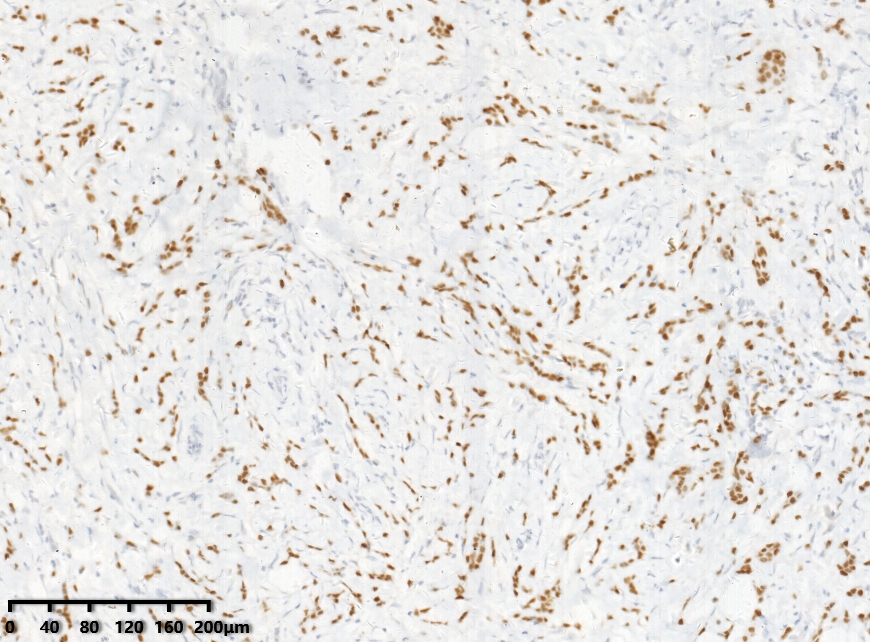

Supplement: Supplementary file 3 [file DataSheet1.zip › immunohistochemistry pictures of breast ILC/ER 100x.jpg]

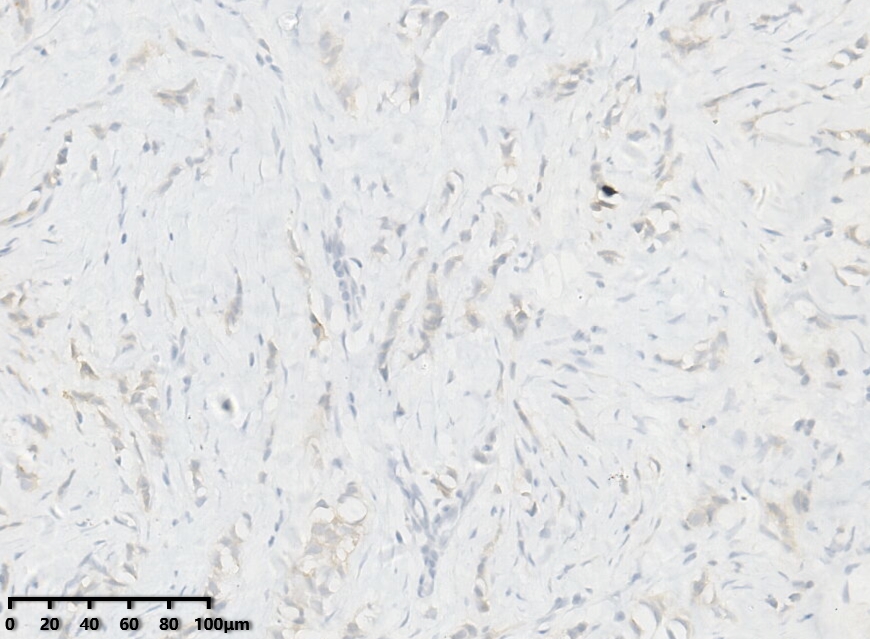

Supplement: Supplementary file 3 [file DataSheet1.zip › immunohistochemistry pictures of breast ILC/HER2 200x.jpg]

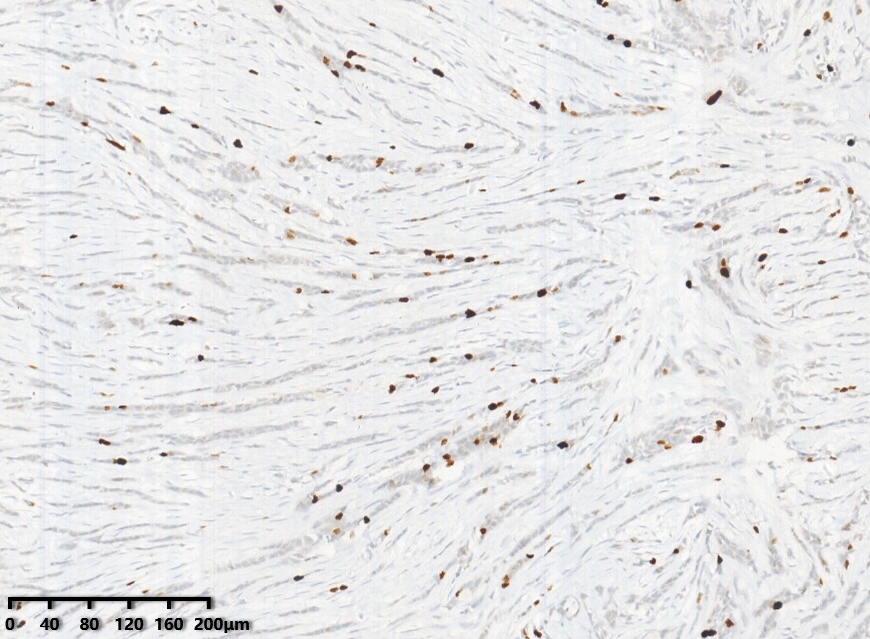

Supplement: Supplementary file 3 [file DataSheet1.zip › immunohistochemistry pictures of breast ILC/ki67 100x.jpg]

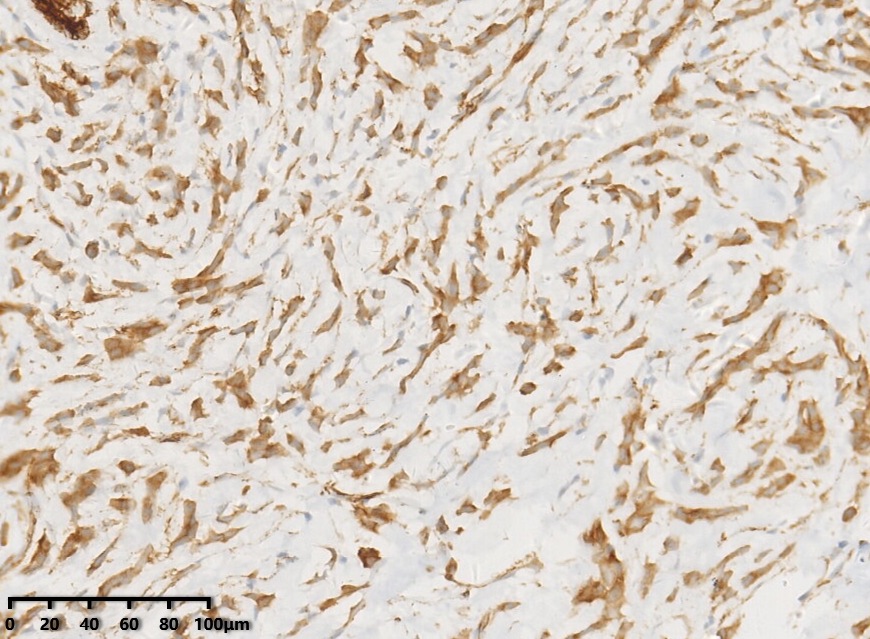

Supplement: Supplementary file 3 [file DataSheet1.zip › immunohistochemistry pictures of breast ILC/P120 200x.jpg]

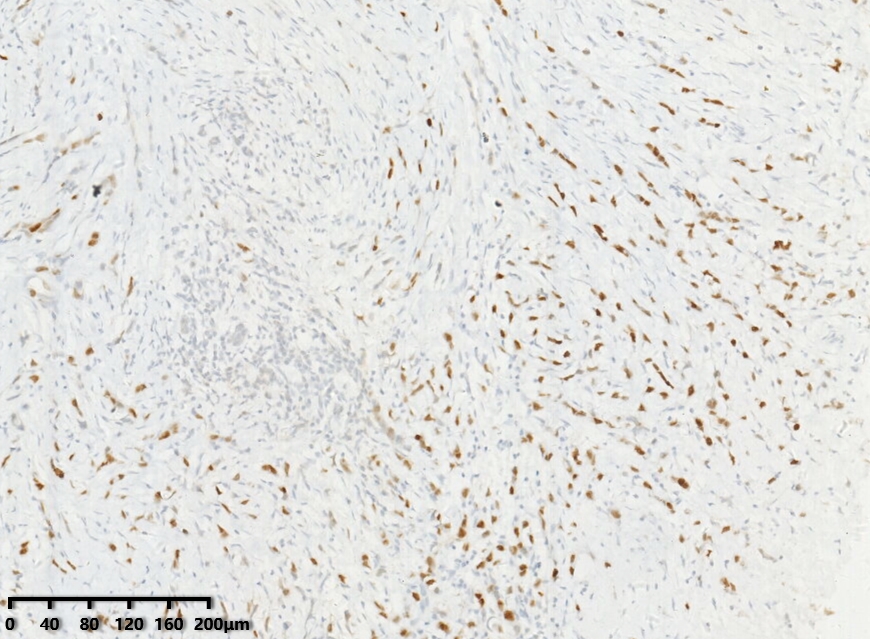

Supplement: Supplementary file 3 [file DataSheet1.zip › immunohistochemistry pictures of breast ILC/PR 100x.jpg]

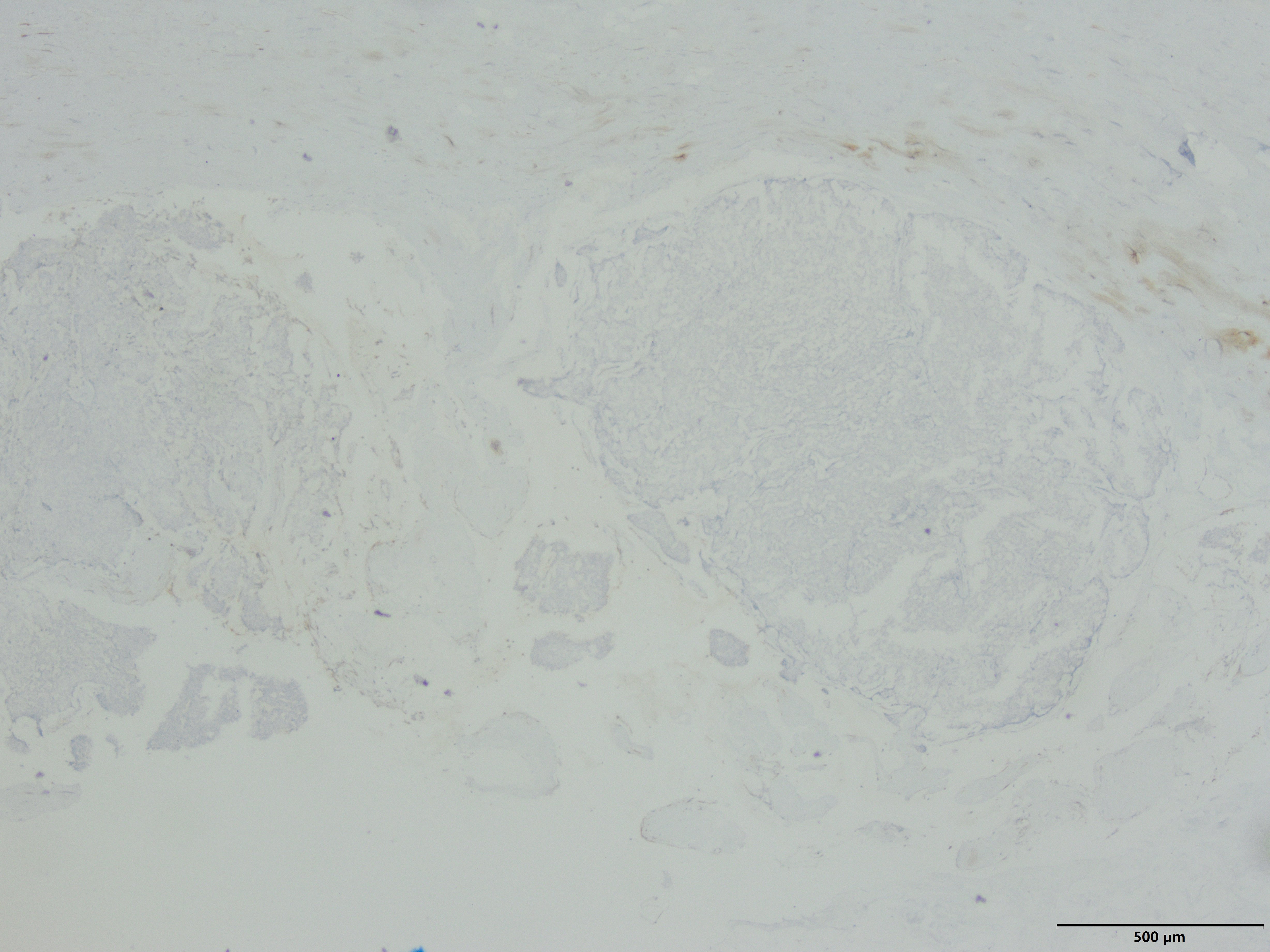

Supplement: Supplementary file 4 [file DataSheet2.zip › immunohistochemistry pictures of breast SPC/Calponin 40x.jpg]

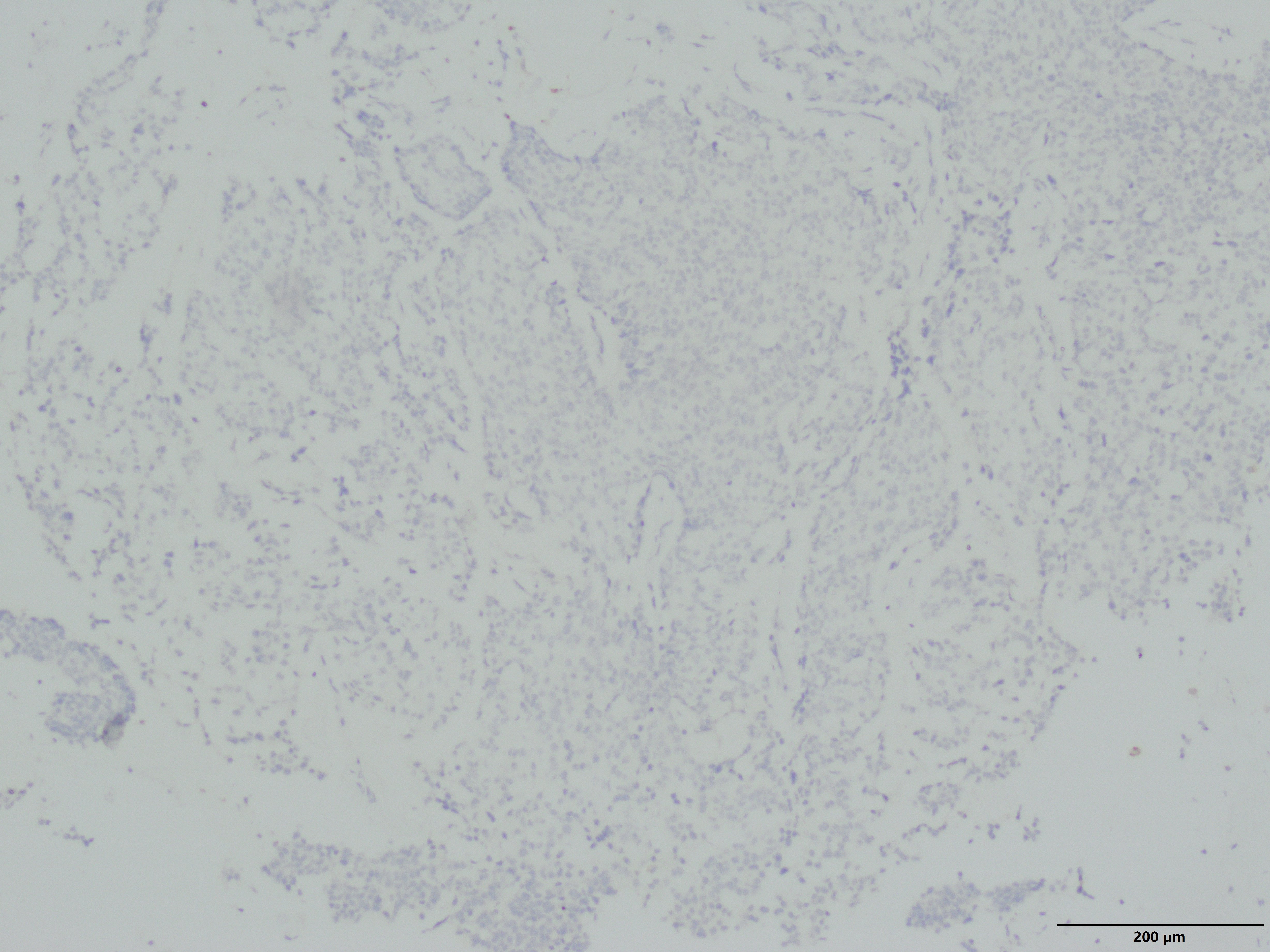

Supplement: Supplementary file 4 [file DataSheet2.zip › immunohistochemistry pictures of breast SPC/CgA 100x.jpg]

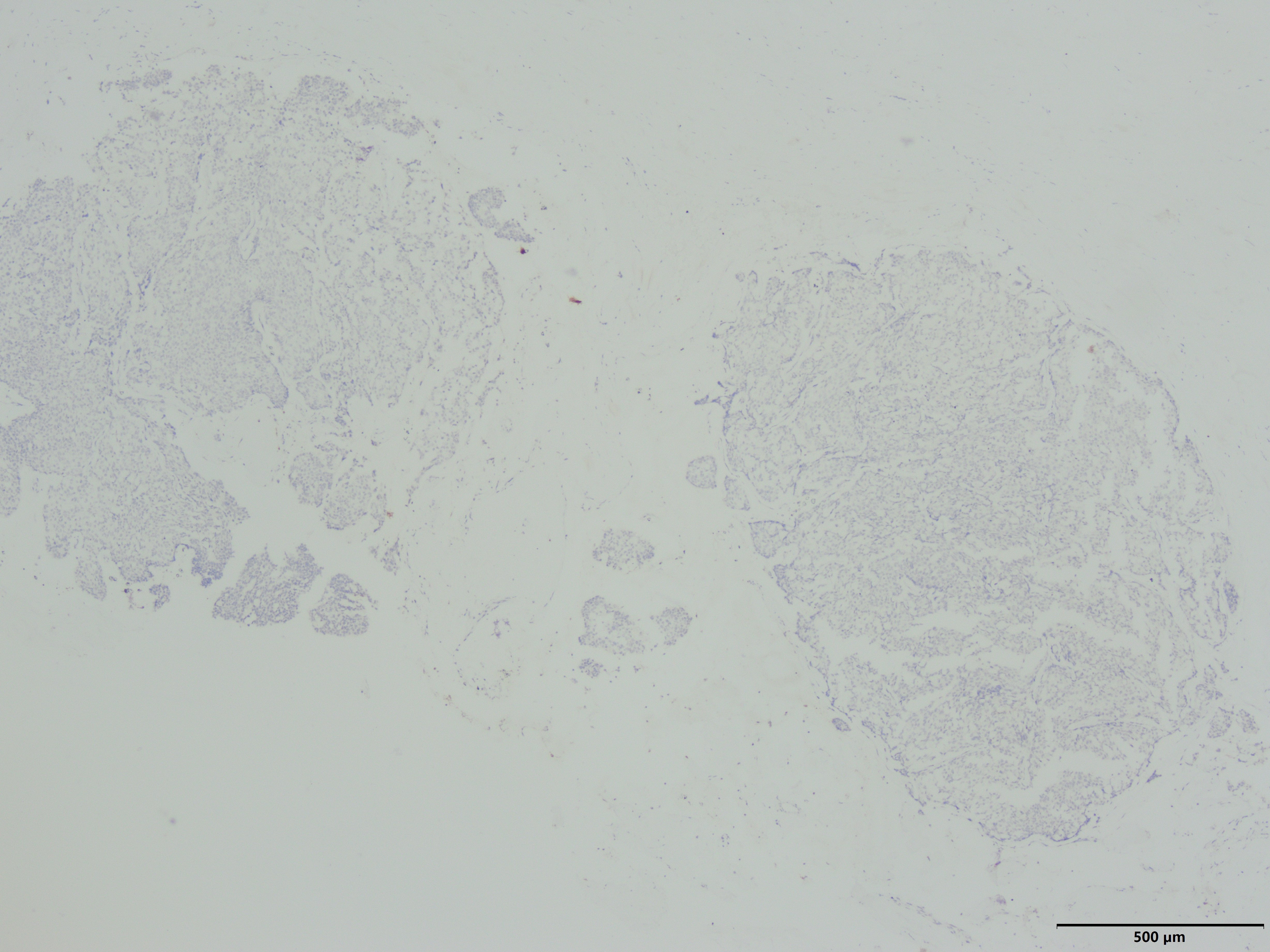

Supplement: Supplementary file 4 [file DataSheet2.zip › immunohistochemistry pictures of breast SPC/CK5&6 40x.jpg]

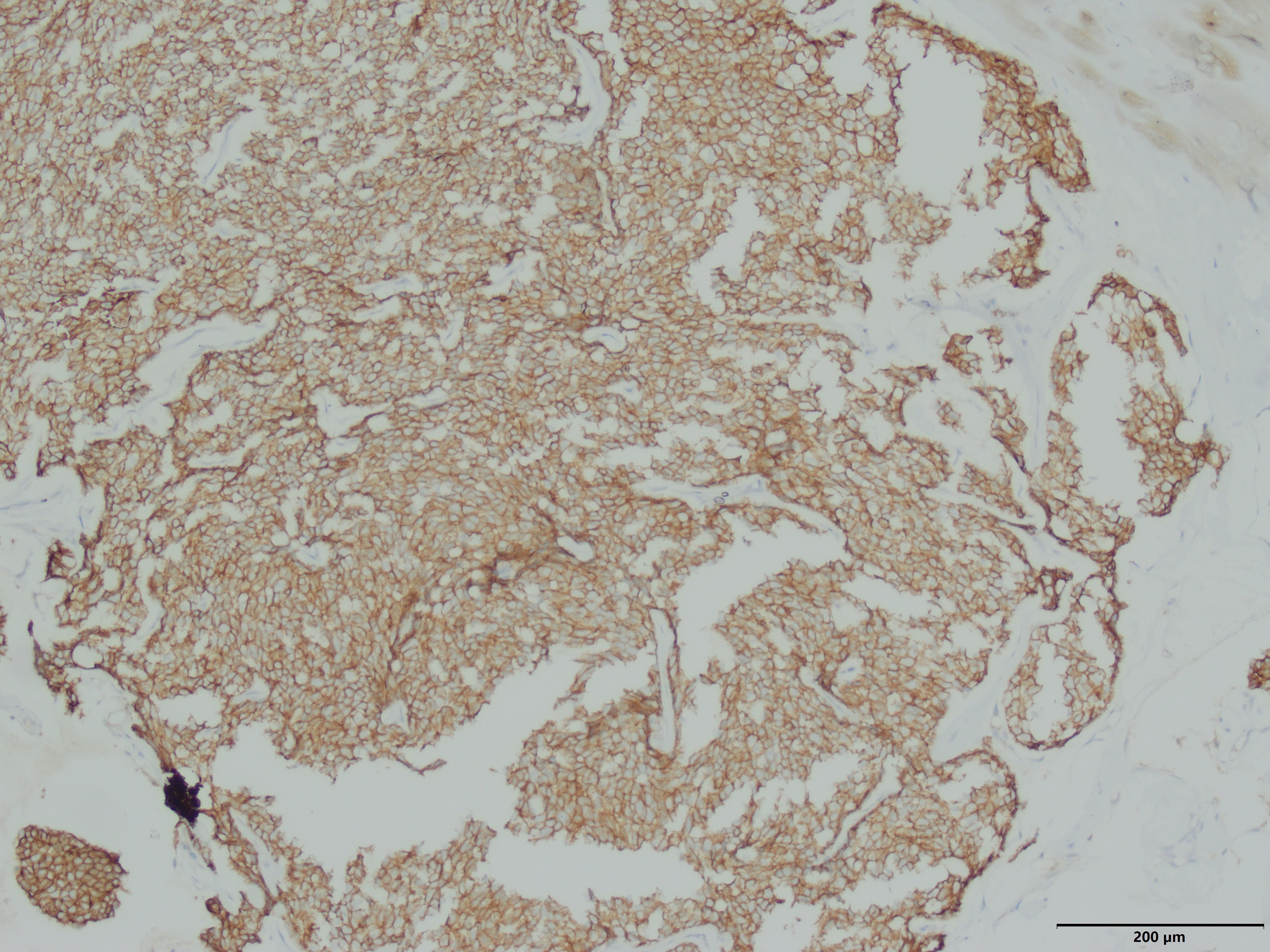

Supplement: Supplementary file 4 [file DataSheet2.zip › immunohistochemistry pictures of breast SPC/E-cadherin 100x.jpg]

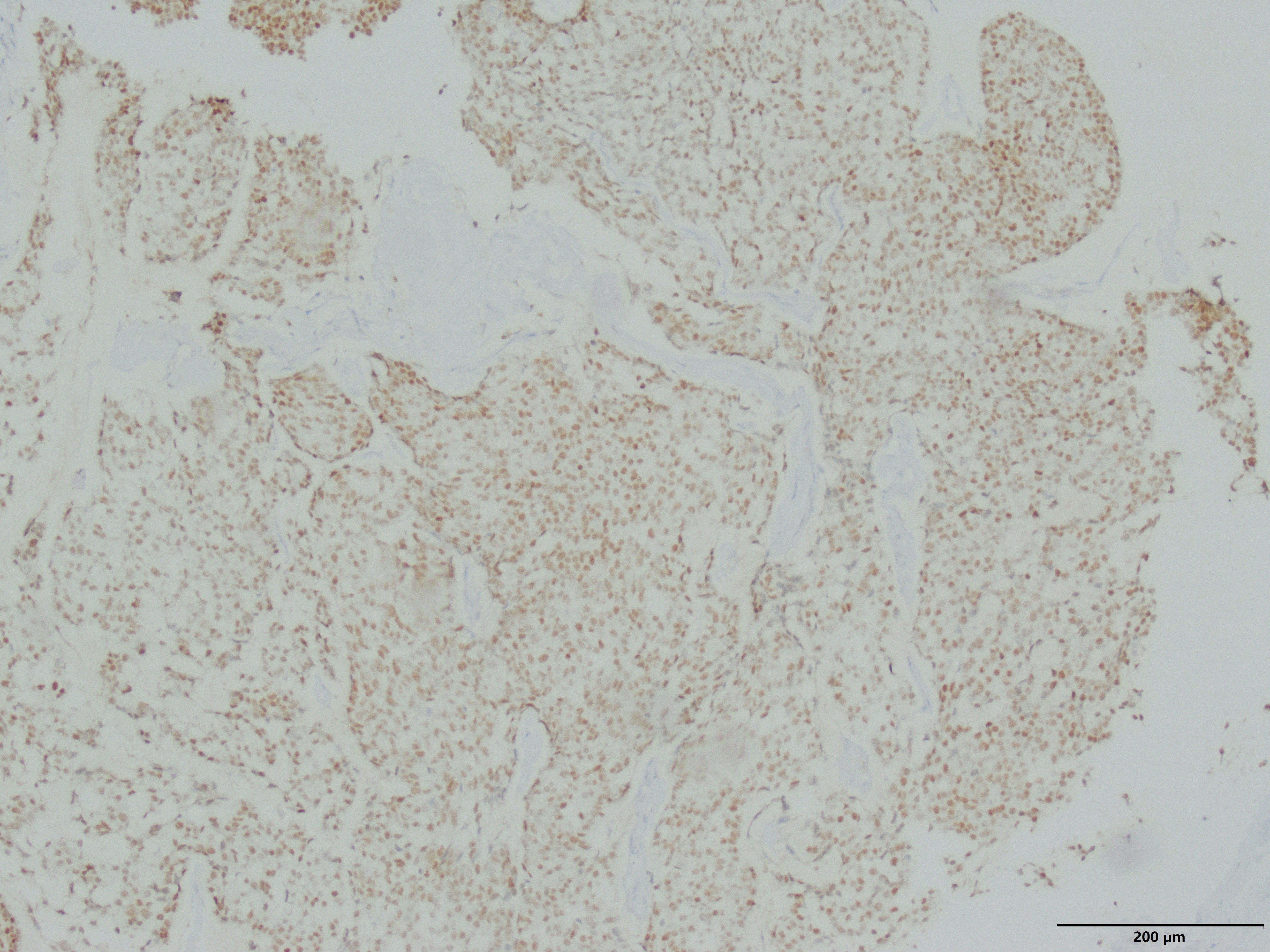

Supplement: Supplementary file 4 [file DataSheet2.zip › immunohistochemistry pictures of breast SPC/ER 100x.jpg]

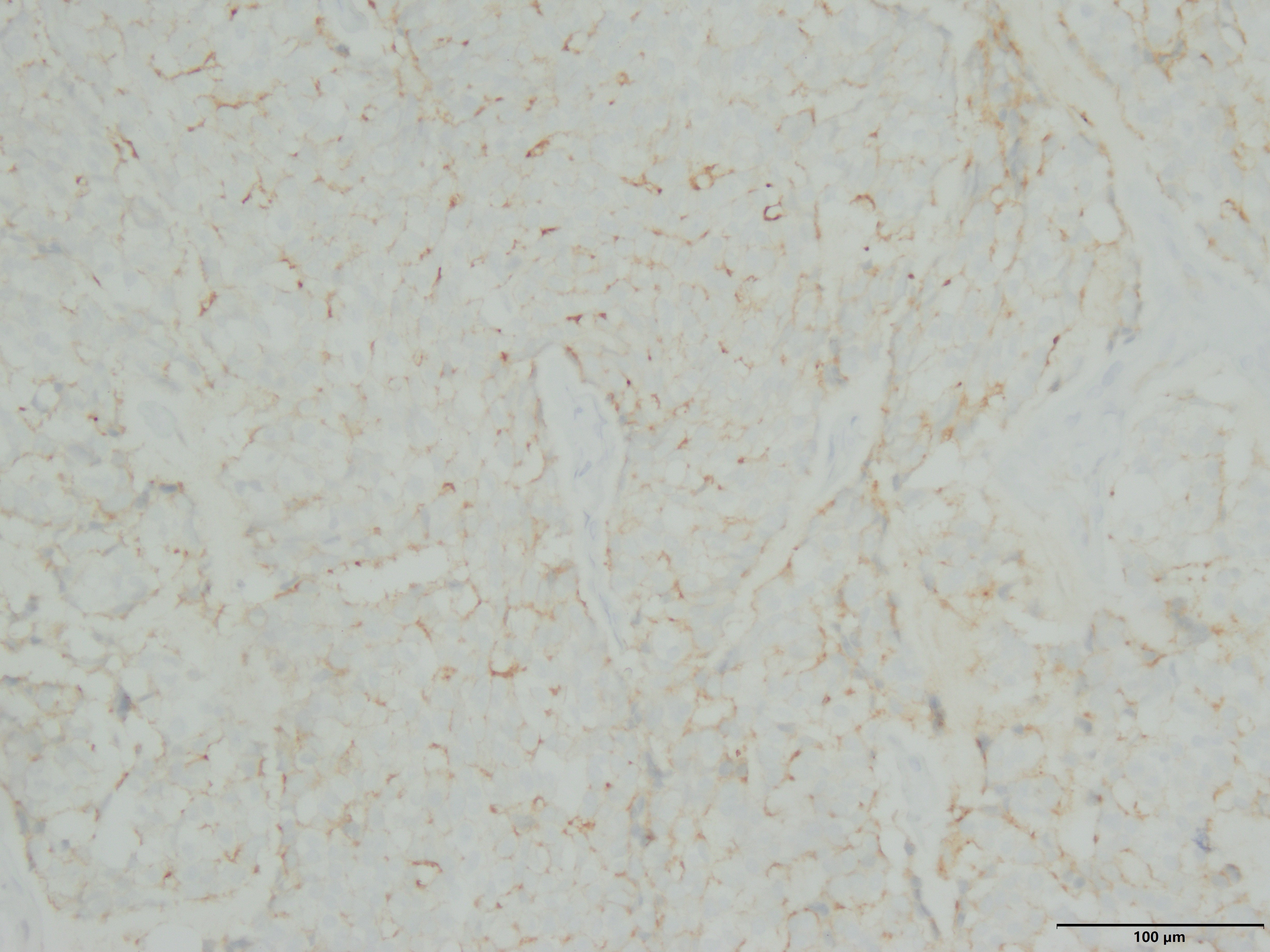

Supplement: Supplementary file 4 [file DataSheet2.zip › immunohistochemistry pictures of breast SPC/HER2 200x.jpg]

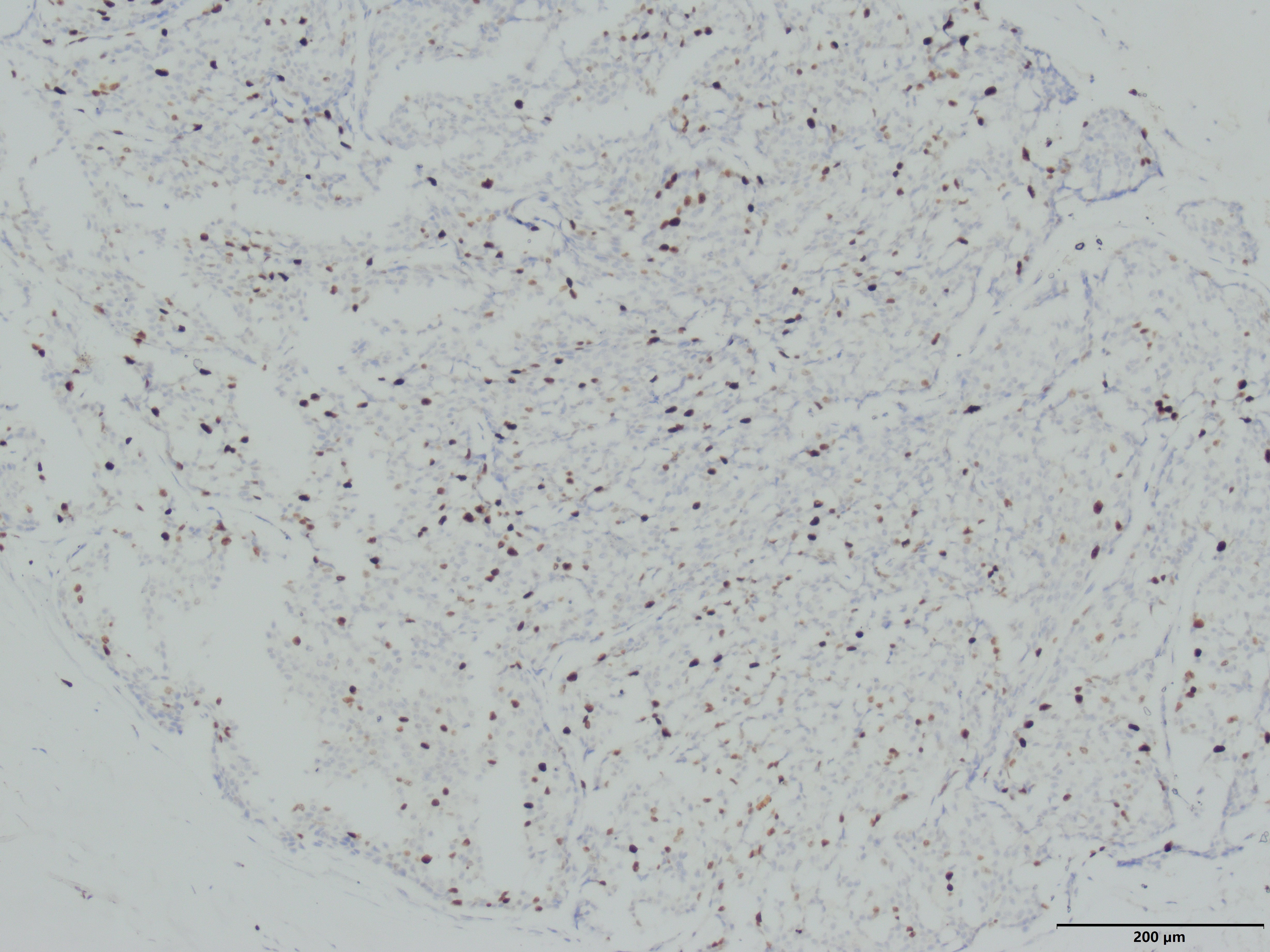

Supplement: Supplementary file 4 [file DataSheet2.zip › immunohistochemistry pictures of breast SPC/Ki67 100x.jpg]

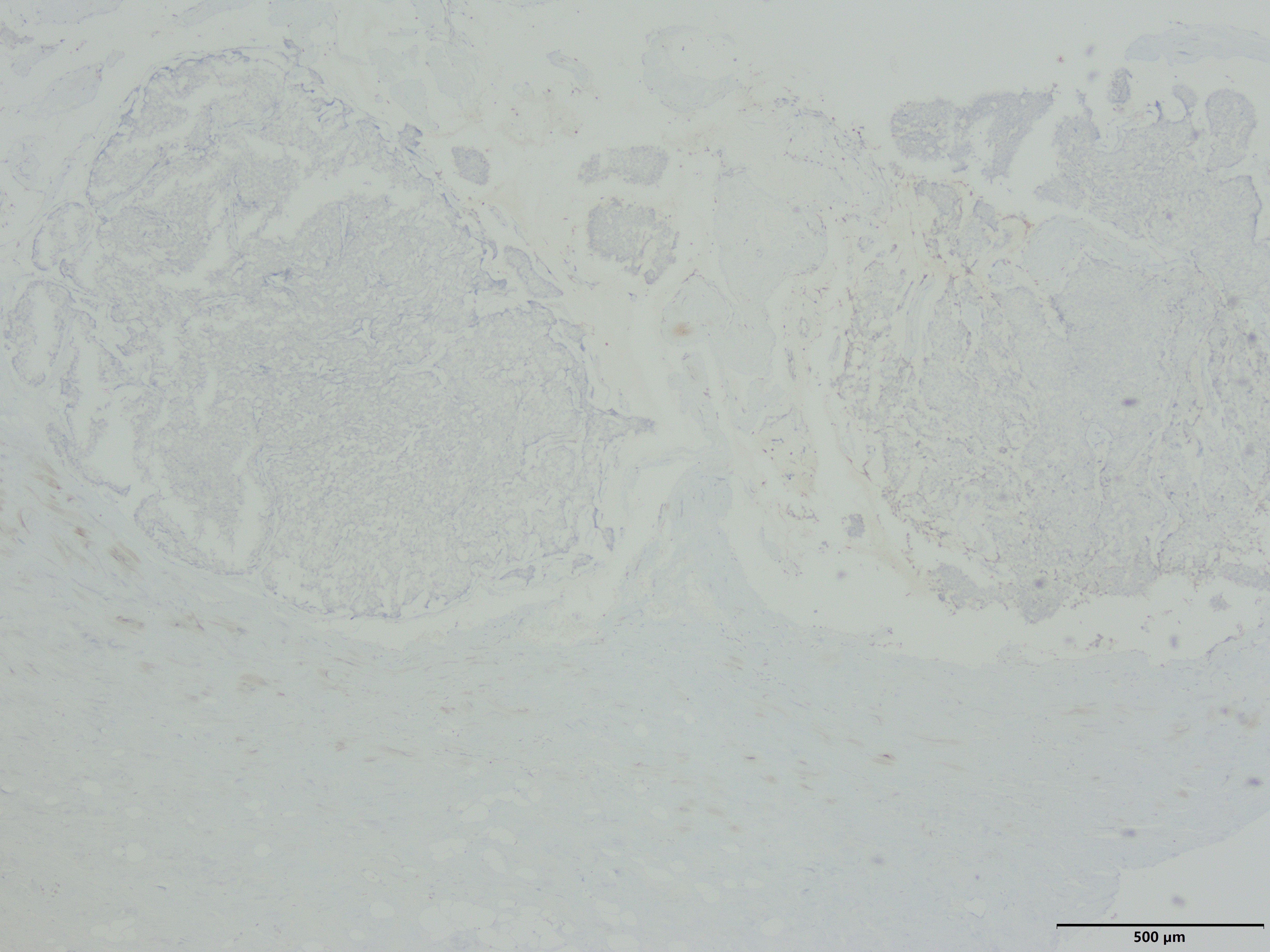

Supplement: Supplementary file 4 [file DataSheet2.zip › immunohistochemistry pictures of breast SPC/P63 40x.jpg]

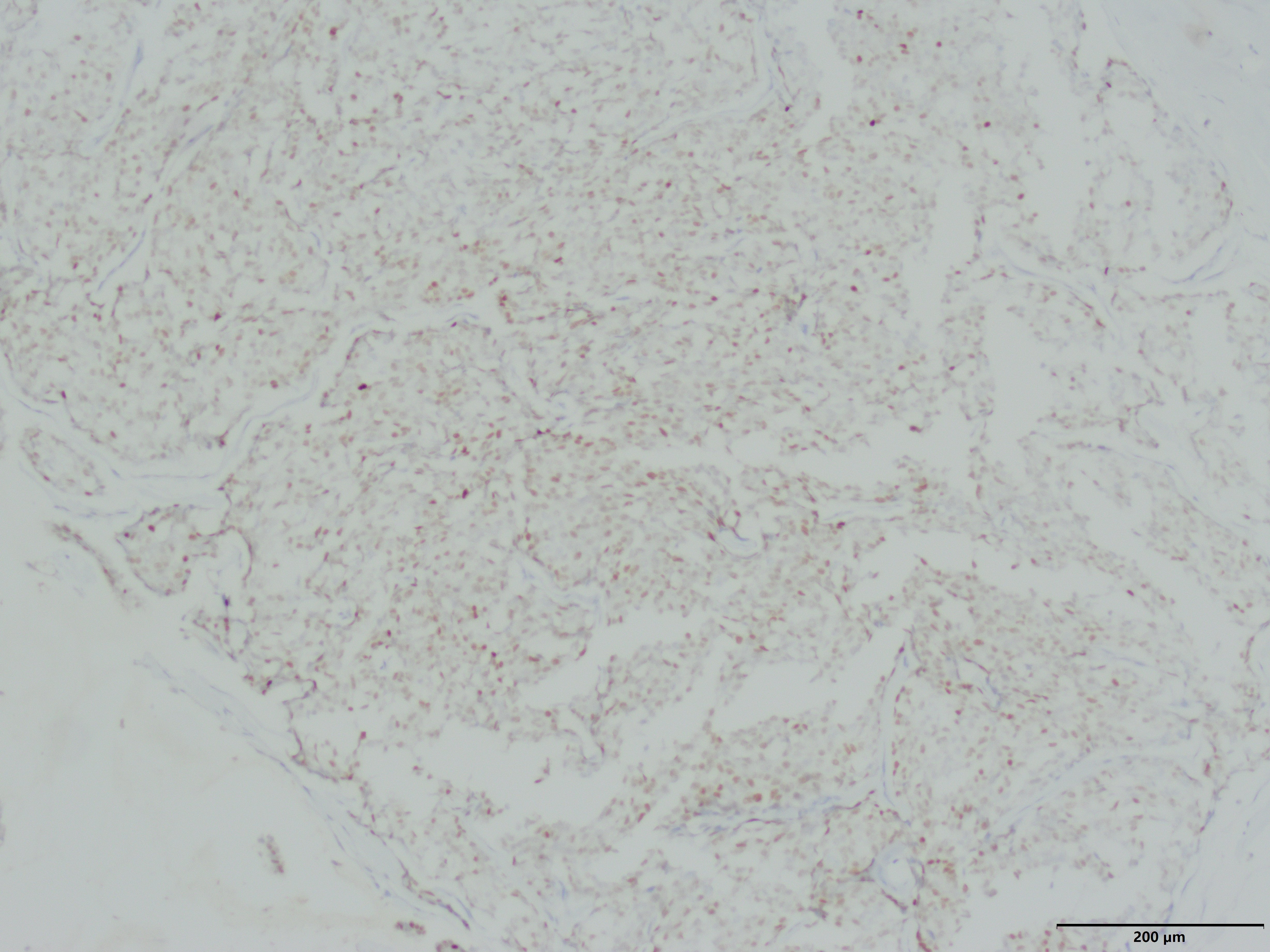

Supplement: Supplementary file 4 [file DataSheet2.zip › immunohistochemistry pictures of breast SPC/PR 100x.jpg]

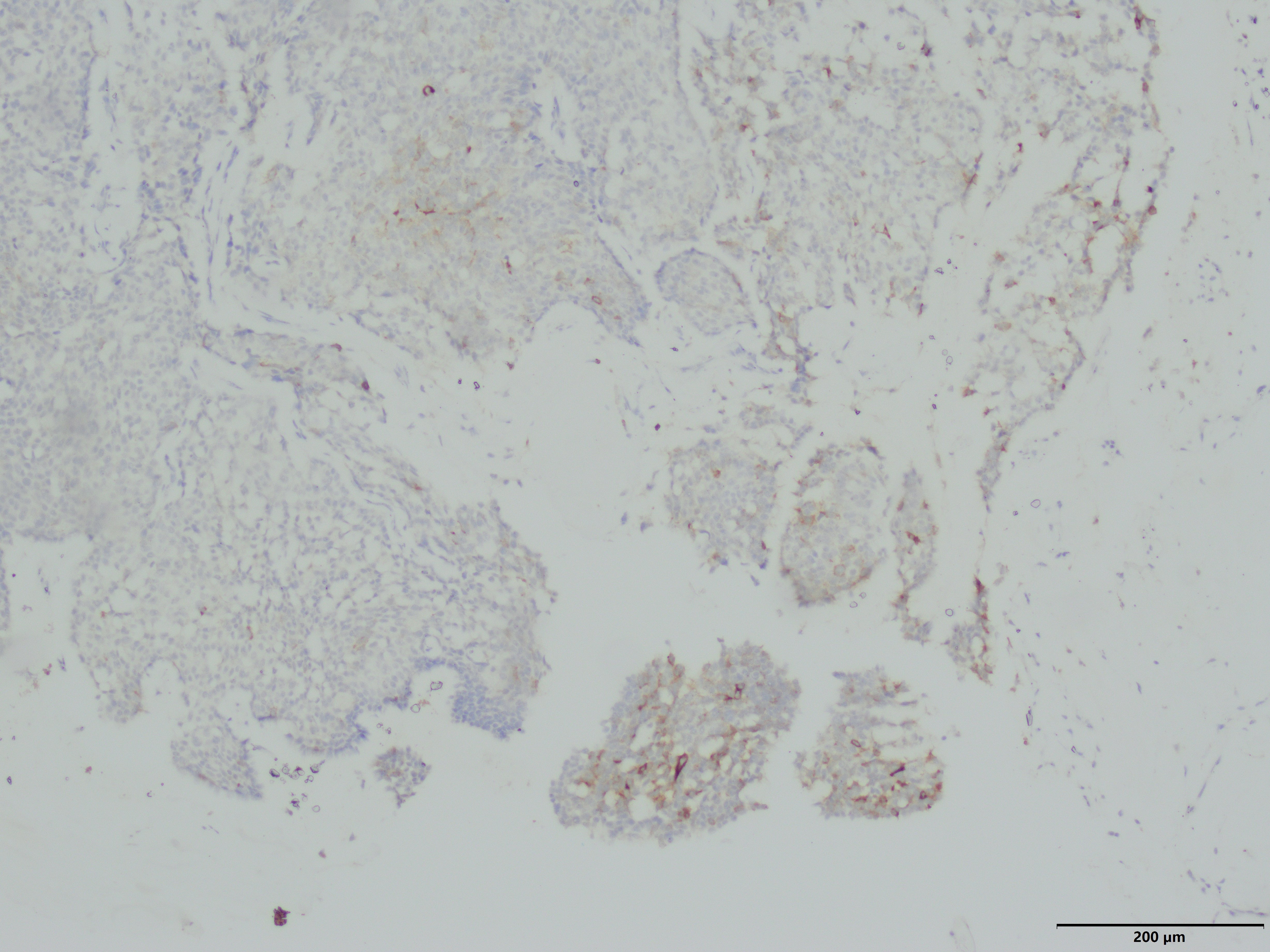

Supplement: Supplementary file 4 [file DataSheet2.zip › immunohistochemistry pictures of breast SPC/Syn 100x.jpg]

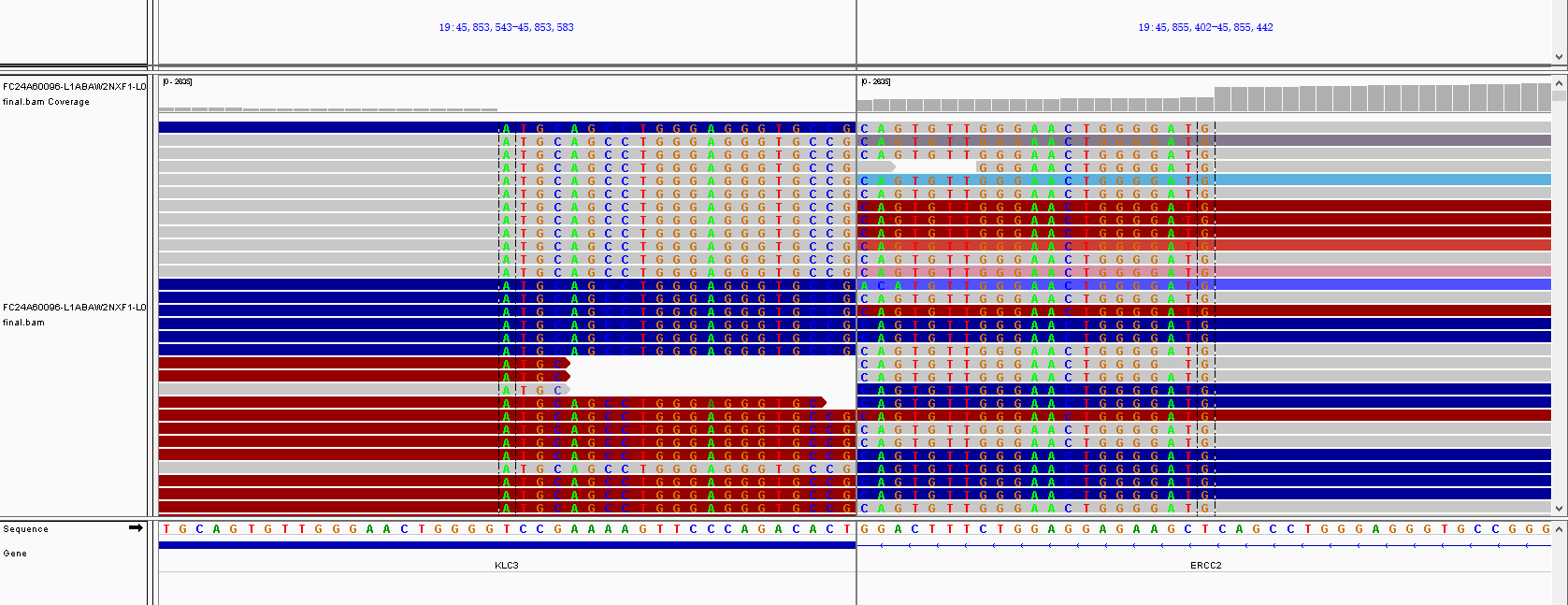

Supplement: Supplementary file 5 [file Image1.png]

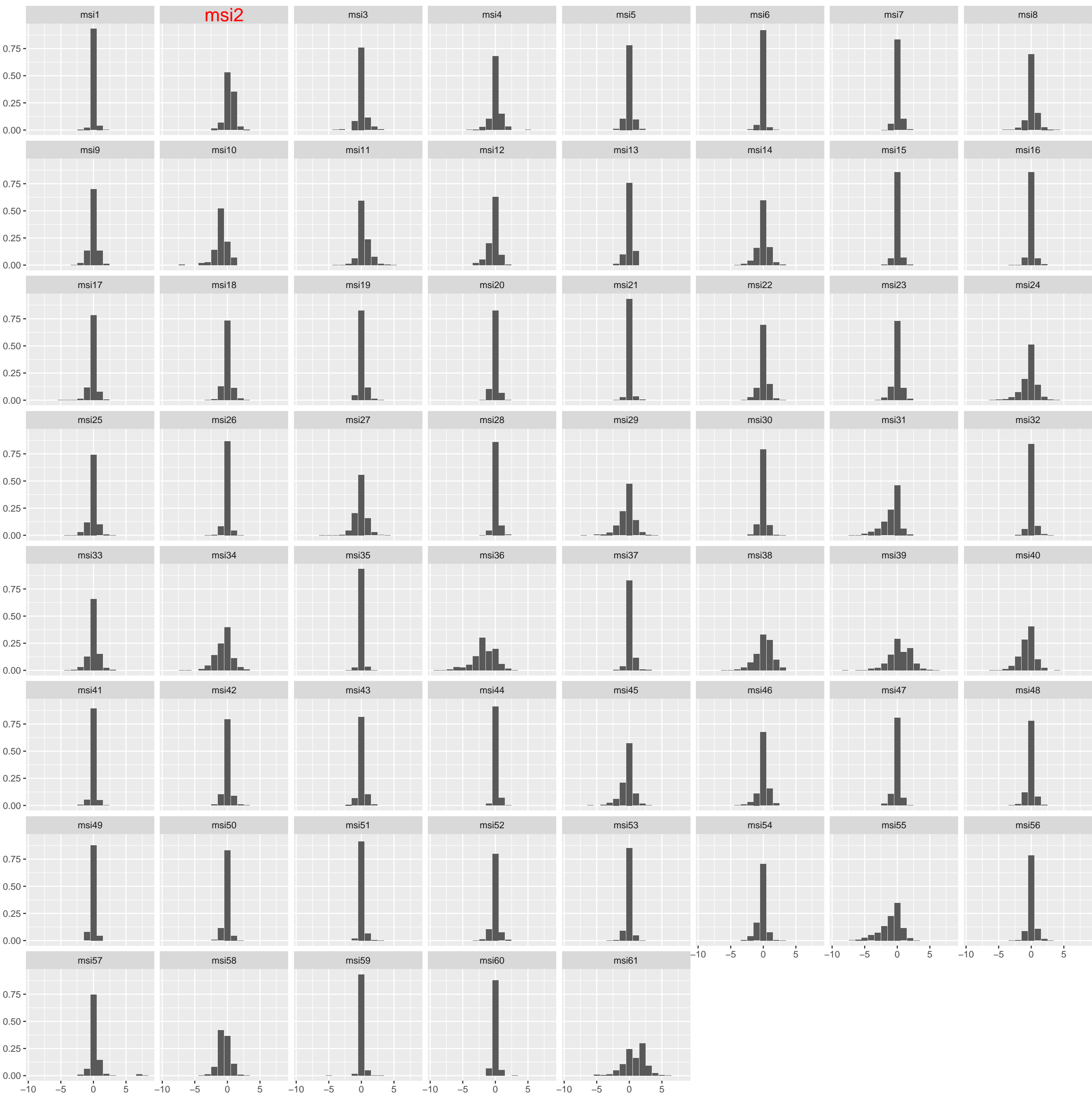

Supplement: Supplementary file 6 [file DataSheet5.zip › Raw data of NGS FC24A60096-L1ABAW2NXF1-L000J124/FC24A60096-L1ABAW2NXF1-L000J124/FC24A60096-L1ABAW2NXF1-L000J124.msisensor2.pdf]

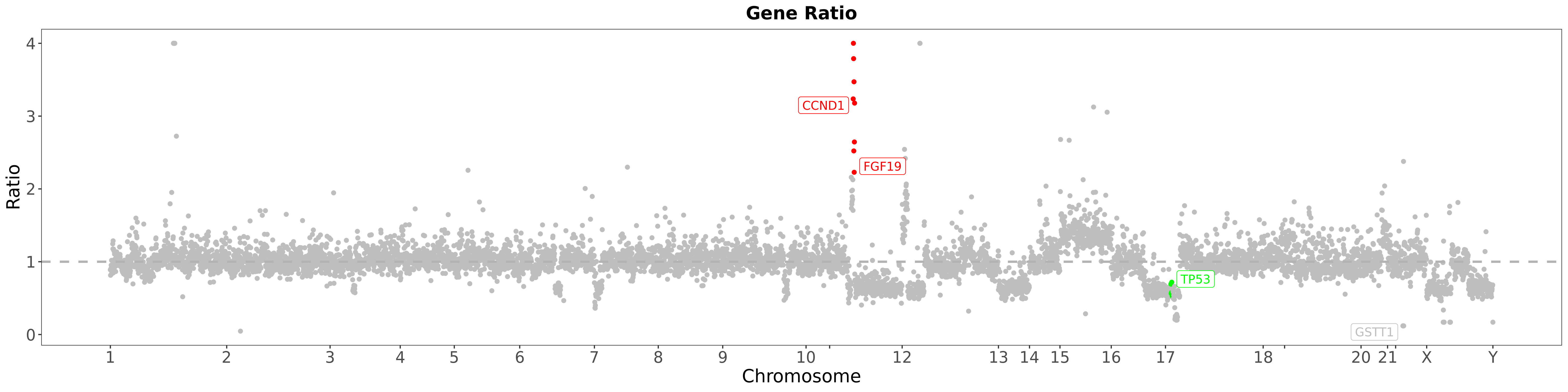

Supplement: Supplementary file 6 [file DataSheet5.zip › Raw data of NGS FC24A60096-L1ABAW2NXF1-L000J124/FC24A60096-L1ABAW2NXF1-L000J124/FC24A60096-L1ABAW2NXF1-L000J124_cnv_gene.png]

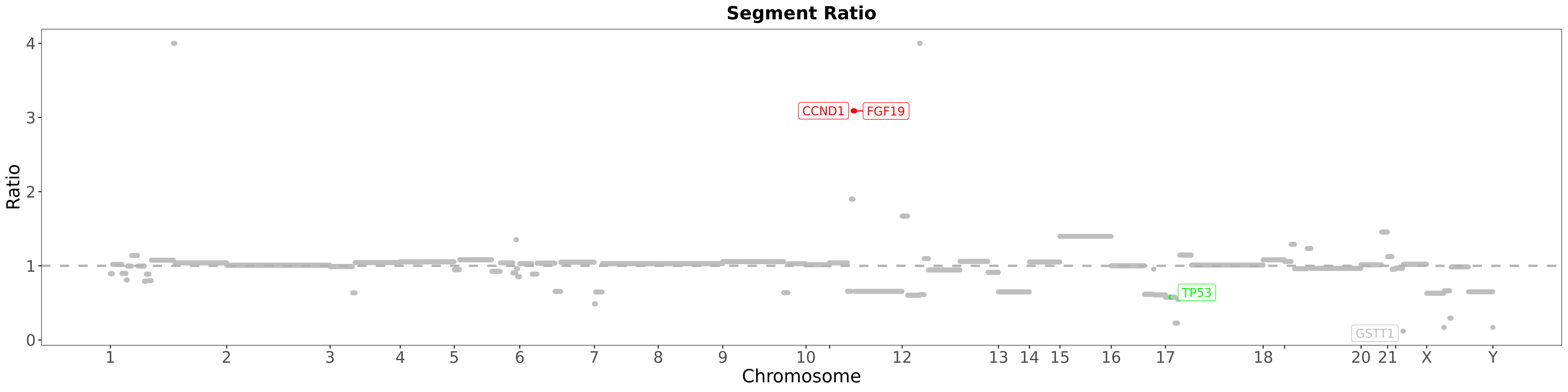

Supplement: Supplementary file 6 [file DataSheet5.zip › Raw data of NGS FC24A60096-L1ABAW2NXF1-L000J124/FC24A60096-L1ABAW2NXF1-L000J124/FC24A60096-L1ABAW2NXF1-L000J124_cnv_plot.png]
